# Designed Inhibitors of Insulin-Degrading Enzyme Regulate the Catabolism and Activity of Insulin

**DOI:** 10.1371/journal.pone.0010504

**Published:** 2010-05-07

**Authors:** Malcolm A. Leissring, Enrico Malito, Sabrine Hedouin, Lael Reinstatler, Tomoko Sahara, Samer O. Abdul-Hay, Shakeel Choudhry, Ghulam M. Maharvi, Abdul H. Fauq, Malwina Huzarska, Philip S. May, Sungwoon Choi, Todd P. Logan, Benjamin E. Turk, Lewis C. Cantley, Marika Manolopoulou, Wei-Jen Tang, Ross L. Stein, Gregory D. Cuny, Dennis J. Selkoe

**Affiliations:** 1 Department of Neuroscience, Mayo Clinic Florida, Jacksonville, Florida, United States of America; 2 Department of Molecular Therapeutics, The Scripps Research Institute, Scripps Florida, Jupiter, Florida, United States of America; 3 Center for Neurologic Diseases, Brigham & Women's Hospital and Harvard Medical School, Boston, Massachusetts, United States of America; 4 Ben-May Institute for Cancer Research, University of Chicago, Chicago, Illinois, United States of America; 5 Laboratory for Drug Discovery in Neurodegeneration, Brigham & Women's Hospital and Harvard Medical School, Cambridge, Massachusetts, United States of America; 6 Department of Pharmacology, Yale University School of Medicine, New Haven, Connecticut, United States of America; 7 Department of Medicine, Beth Israel Deaconess Medical Center and Harvard Medical School, Boston, Massachusetts, United States of America; Johns Hopkins School of Medicine, United States of America

## Abstract

**Background:**

Insulin is a vital peptide hormone that is a central regulator of glucose homeostasis, and impairments in insulin signaling cause diabetes mellitus. In principle, it should be possible to enhance the activity of insulin by inhibiting its catabolism, which is mediated primarily by insulin-degrading enzyme (IDE), a structurally and evolutionarily distinctive zinc-metalloprotease. Despite interest in pharmacological inhibition of IDE as an attractive anti-diabetic approach dating to the 1950s, potent and selective inhibitors of IDE have not yet emerged.

**Methodology/Principal Findings:**

We used a rational design approach based on analysis of combinatorial peptide mixtures and focused compound libraries to develop novel peptide hydroxamic acid inhibitors of IDE. The resulting compounds are ∼10^6^ times more potent than existing inhibitors, non-toxic, and surprisingly selective for IDE *vis-à-vis* conventional zinc-metalloproteases. Crystallographic analysis of an IDE-inhibitor complex reveals a novel mode of inhibition based on stabilization of IDE's “closed,” inactive conformation. We show further that pharmacological inhibition of IDE potentiates insulin signaling by a mechanism involving reduced catabolism of internalized insulin.

**Conclusions/Significance:**

The inhibitors we describe are the first to potently and selectively inhibit IDE or indeed any member of this atypical zinc-metalloprotease superfamily. The distinctive structure of IDE's active site, and the mode of action of our inhibitors, suggests that it may be possible to develop inhibitors that cross-react minimally with conventional zinc-metalloproteases. Significantly, our results reveal that insulin signaling is normally regulated by IDE activity not only extracellularly but also within cells, supporting the longstanding view that IDE inhibitors could hold therapeutic value for the treatment of diabetes.

## Introduction

Insulin is a tightly regulated peptide hormone that is centrally invovled in multiple vital physiological processes, ranging from energy and glucose homeostasis to memory and cognition [1], [2], [3]. The tertiary structure of insulin is unique among peptide hormones, being comprised of 2 peptide chains and containing 1 intra- and 2 interchain disulfide bonds, and the relative rigidity and bulk of insulin render it a poor substrate for most proteases [4].

The proteolytic degradation and inactivation of insulin is believed to be mediated primarily by insulin-degrading enzyme (IDE), a ubiquitously expressed, soluble, secreted zinc-metalloprotease [5], [6]. IDE belongs to a small superfamily of zinc-metalloproteases (clan ME, family M16) that evolved independently of conventional zinc-metalloproteases [7]. Members of this superfamily are commonly referred to as “inverzincins,” because they feature a zinc-binding motif (HxxEH) that is inverted with respect to that within conventional zinc-metalloproteases (HExxH) [8].

Like insulin, IDE is structurally distinctive, consisting of two bowl-shaped halves connected by a flexible linker that can switch between “open” and “closed” states [9]. In its closed state, IDE completely encapsulates its substrates within an unusually large internal cavity [9] that appears remarkably well-adapted to accommodate insulin [10]. IDE degrades several other intermediate-sized peptides, including atrial natriuric peptide, glucagon, and the amyloid β-protein (Aβ) [11]; however, unlike insulin, most other IDE substrates are known to be hydrolyzed by multiple proteases.

Diabetes melittus is a life-threatening and highly prevalent group of endocrinological disorders that, fundamentally, are characterized by impaired insulin signaling. Correspondingly, it is the common goal of most anti-diabetic therapies to enhance insulin signaling, either by direct injection of insulin, by stimulating the production or secretion of endogenous insulin, or by activating downstream targets of the insulin receptor (IR) signaling cascade [12]. In principle, it should be possible to enhance insulin signaling by inhibiting IDE-mediated insulin catabolism [13]. Pharmacological inhibitors of IDE in fact attracted considerable attention in the decades following the discovery of IDE in 1949 [14]. Quite significantly, a purified inhibitor of IDE (of undetermined identity) was found to potentiate the hypoglycemic action of insulin *in vivo* as early as 1955 [15].

Despite more than 60 years of research on IDE and its involvement in insulin catabolism, the development of small-molecule inhibitors of IDE has proved to be a surprisingly elusive goal [16]. We describe herein the design, synthesis, enzymologic characterization, and enzyme-bound crystal structure of the first potent and selective inhibitors of IDE. In addition, we show that inhibition of IDE can potentiate insulin signaling within cells, by reducing the catabolism of internalized insulin. These novel IDE inhibitors represent important new pharmacological tools for the experimental manipulation of IDE and, by extension, insulin signaling. Furthermore, our results lend new support to the old idea that pharmacological inhibition of IDE may represent an attractive approach to the treatment of diabetes mellitus.

## Results

### Compound screening fails to identify effective IDE inhibitors

The few IDE inhibitors currently in use are generally non-selective and/or highly toxic, and all suffer from extremely low potency, requiring mM concentrations to achieve complete inhibition (Table S1). To develop improved inhibitors of IDE, we initially conducted high-throughput screening on ∼115,000 compounds, using a previously developed Aβ degradation assay [17]. Although several inhibitors were discovered, most of these were either toxic, thiol-alkylating compounds [18] (Fig. S1A) or were compounds acting through other mechanisms that proved difficult to improve despite extensive medicinal chemistry optimization efforts (Fig. S1B). Among the remaining inhibitors, just a single compound, known as nullscript [19] (Fig. S1C), exhibited submicromolar IC_50_ values.

Nullscript is predicted to target the active site of IDE because it contains the potent hydroxamic acid zinc-binding moiety, but it proved ineffective in cell-based assays (not shown). Consequently, we tested a range of peptidic and nonpeptidic hydroxamate inhibitors of conventional metalloproteases for possible effects on IDE. Surprisingly, IDE was unaffected by any of the nonpeptidic hydroxamates tested, some of which were potent inhibitors of a broad range of zinc-metalloproteases, and was only weakly inhibited by peptidic versions (Table S2). This result provides a striking confirmation that IDE's active site—though obviously optimized to accommodate peptides—is indeed substantially different from the active site within canonical zinc-metalloproteases, raising the possibility that highly selective zinc-binding inhibitors might be developed.

### Potent IDE inhibitors developed using a classic design strategy

**Figure 1:**
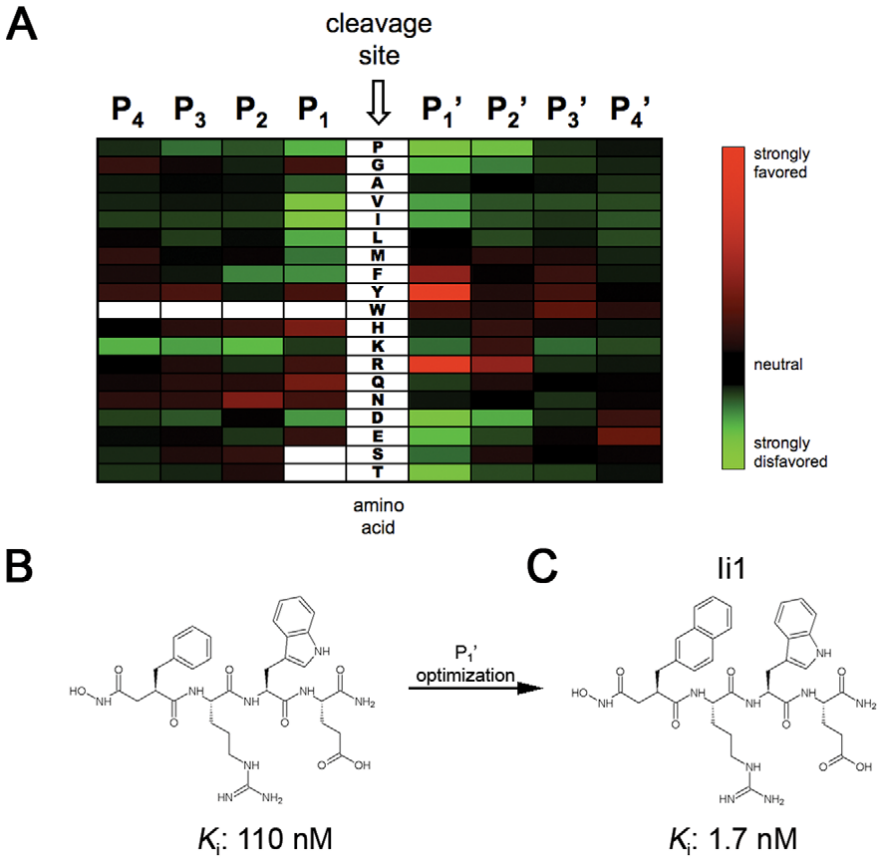
Cleavage-site specificity of IDE and first-generation inhibitors derived therefrom. ***A***, Heat map showing amino acids favored (*red*) or disfavored (*green*) by IDE at different positions relative to the cleavage site, as determined from proteomic analysis of peptide mixtures. ***B***, Conventional peptide hydroxamate synthesized on the basis of the analysis in (**A**). ***C***, Structure of inhibitor Ii1, incorporating optimized P_1_' moiety deduced from analysis of a focused library of retro-inverso peptide hydroxamates (see Figure 2).

These initial results prompted us to revisit the classic design strategy originally used to develop zinc-metalloprotease inhibitors [20]. The general approach is to attach a zinc-binding moiety to short peptides that correspond to the cleavage-site specificity of the protease of interest [21]. Because IDE lacks a clear cleavage-site specificity deducible from known substrates, we determined this *de novo* through proteomic analysis of the cleavage sites within combinatorial mixtures of short peptides (see *Methods*) [22]. This analysis revealed that IDE has a strong preference for Tyr≈Arg > Phe at the P_1_' position and for Arg at the P_2_' position, while showing relatively little specificity at the remaining sites (Fig. 1A).

**Figure 2:**
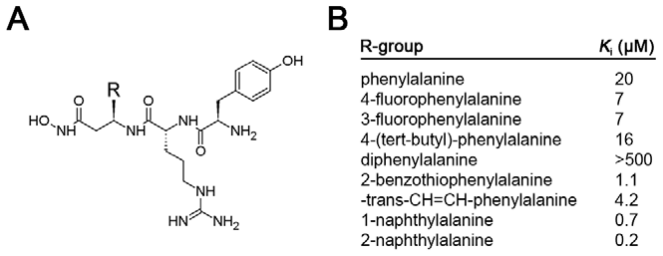
IDE inhibition by a focused library of retro-inverso peptide hydroxamates containing modifications at the P_1_' position. ***A***, Structure of compound library. Note that compounds were generated in the retro-inverso configuration (see Fig. S2). ***B***, Relative *K*_i_ values of inhibitors containing different R groups (see **A**).

Based on this analysis and considerations of synthetic tractability, we initially generated inhibitors based on the peptide Phe-Arg-Trp-Glu. A hydroxamic acid moiety was attached to the N-terminus, positioned appropriately by the inclusion of an α carbonyl moiety [21]. We generated pure diastereomers containing either all L-amino acids or a D-amino acid at the P_1_' position, yielding *K*_i_ values of 0.11 (Fig. 1B) and 4.1 µM, respectively. While these compounds are far more potent than previously described IDE inhibitors, their potency is poorer than is typically achieved with peptide hydroxamates, and they proved non selective when tested against other metalloproteases (not shown). We postulated that these deficiencies were related to the incorporation of Phe at the P_1_' position, which was not as strongly or uniquely preferred as Tyr or Arg (Fig. 1A). To optimize the P_1_' position, we generated a focused library of peptide hydroxamates based on the sequence Xaa-Arg-Tyr, where Xaa represented a variety of natural and unnatural amino acids (Fig. 2A). These peptides were generated by solid-phase peptide synthesis in the “retro-inverso” configuration, wherein the peptide backbone is reversed with respect to conventional peptides (see Fig. S2). The retro-inverso configuration required the use of all D-amino acids, and the need to incorporate an additional α carbon between the hydroxamic acid and the peptide sequence required the use of β-amino acids at the P_1_' position. A ∼100-fold gain in potency was realized when Phe was substituted with 2-naphthylalanine (2-Nap; Fig. 2B). We then generated a conventional peptide hydroxamate incorporating 2-Nap at the P_1_' position, yielding an inhibitor, Ii1 (IDE inhibitor 1; Fig. 1C), with a *K*_i_ value of ∼1.7 nM, or >10^5^ more potent than IDE inhibitors currently in use (*c.f.*, Table S1).

### Novel enzymological properties and unanticipated selectivity of Ii1

**Figure 3:**
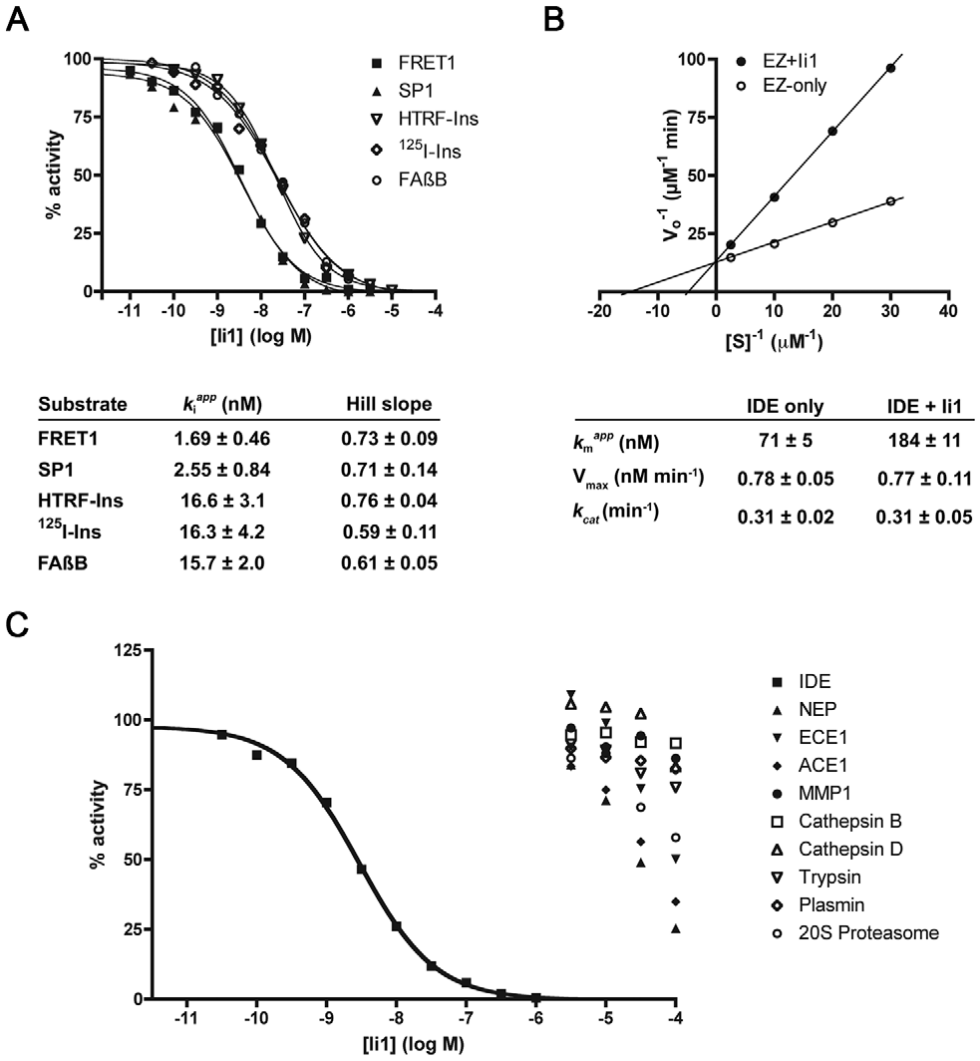
*In vitro* enzymatic analysis of IDE inhibition by Ii1. ***A***, Representative dose-response curves for a variety of IDE substrates and *K*_i_ values and Hill slopes computed from 4 to 6 replications per substrate. Note that [S]/*K*_M_ was kept constant for dose-response studies, permitting visual comparison of relative *K*_i_ values. ***B***, Lineweaver-Burk plot of IDE-mediated insulin degradation in the absence or presence of Ii1 (30 nM) and kinetic parameters calculated from 4 independent experiments. These data were obtained using recombinant human insulin and ^125^I-insulin at a fixed ratio (1000∶1), as described [48]. Note that the mode of inhibition is purely competitive, and was also observed using Aβ (Fig. S3). ***C***, Dose-response curves showing the selectivity of Ii1 for IDE as compared to several other zinc-metalloproteases—neprilysin (NEP), endothelin-converting enzyme-1 (ECE1), angiotensin-converting enzyme-1 (ACE1) and matrix-metalloprotease-1 (MMP1)—and representative members of other protease classes, including cysteine (cathepsin B), aspartate (cathepsin D), serine (trypsin and plasmin) and threonine (20S proteasome). Data are mean of 2 to 3 experiments per condition.

Enzymatic analysis of inhibitor Ii1 (Fig. 3) revealed several unexpected properties. First, the *K*_i_ values computed for Ii1 were found to be substrate dependent, with shorter substrates yielding lower values, and longer substrates—such as insulin and Aβ—yielding values consistently ∼10-fold higher (Fig. 3A). It is notable that a similar pattern of substrate dependence has consistently been observed for pharmacological activators of IDE [23] (see *Discussion*). In agreement with this result, Ii1 exhibited a purely competitive mode of inhibition of the hydrolysis of insulin (Fig. 3B) and Aβ (Fig. S3). Second, the Hill slopes for inhibition of all substrates tested were <1, suggesting some degree of negative cooperativity. Finally, and rather unexpectedly for this type of inhibitor, Ii1 proved to be remarkably selective for IDE *vis-à-vis* a wide range of conventional zinc-metalloproteases and representative members of other protease classes (Fig. 3C).

### Crystal structure of IDE-Ii1 complex reveals a novel mode of inhibition

**Figure 4:**
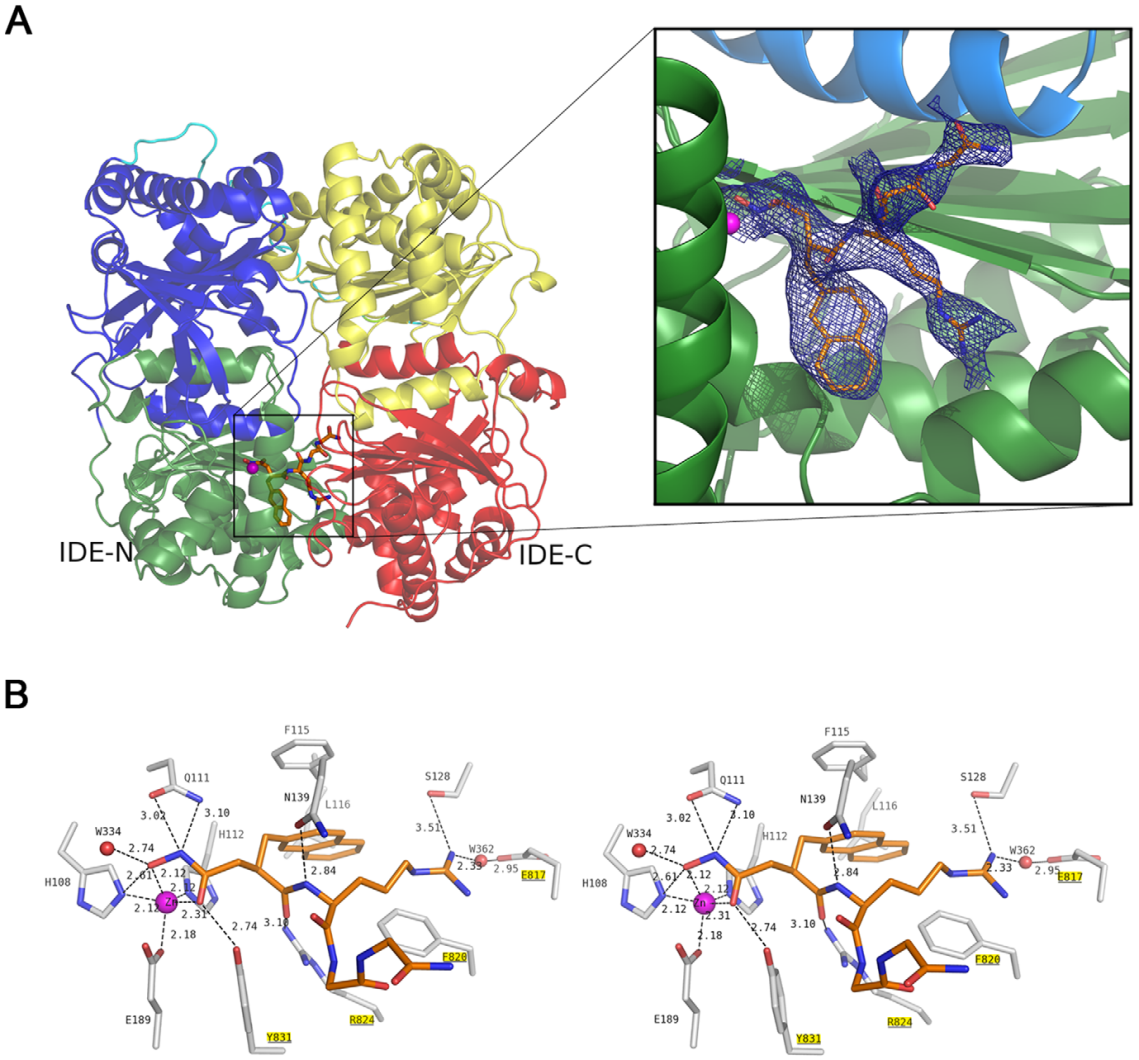
Structure of human IDE complexed to inhibitor Ii1. ***A***, Overall structure of Ii1-bound IDE. Ribbon diagram of monomeric IDE colored as *green*, *blue*, *yellow* and *red* for domains 1, 2, 3 and 4, respectively (*left*). The connecting loop between IDE-N and IDE-C is shown in *cyan*. 2*F_o_–F_c_* simulated annealing omit map contoured at 1 σ is shown in *blue* around Ii1 (*right*). In this view, the C-terminal domains of IDE (*yellow*, *red*) have been omitted to highlight the interactions of Ii1 with the catalytic zinc atom in the N-terminal domain of IDE. ***B***, Stereodiagram showing the detailed interactions of Ii1 with residues and zinc ion in the catalytic site of IDE. Protein residues involved in catalysis and in contact with Ii1, as well as the inhibitor are drawn in *stick representation*. Residues from the C-terminal half of IDE are *underlined* and highlighted in *yellow*. Ii1 in stick representation is colored the same as in (**A**). Carbon nitrogen and oxygen atoms of Ii1 are colored *orange* and those of IDE *grey*; nitrogen and oxygen atoms are colored *blue* and *red*, respectively; zinc and water are represented by *spheres* colored *magenta* and *red*, respectively. Hydrogen bonds are shown as *dashed lines*. Figures generated using Pymol [50].

To elucidate the mechanistic basis for these intriguing properties, we solved the co-crystal structure of Ii1 complexed to catalytically inactive, cysteine-free human IDE at 2.6-Å resolution (Fig. 4; Fig. S4; Table S3). The mode of binding of Ii1 is remarkable in several key respects, which have attendant implications for IDE's mechanism of catalysis and for future drug design. First, the IDE-inhibitor interaction is restricted to the catalytic zinc and subsites S_1_' and S_2_' (Fig. 4A). The P_3_' and P_4_' residues of Ii1 face the center of the catalytic chamber of IDE and do not contact IDE directly; consequently, only the main chain of these two residues could be modeled (Fig. 4B). This is consistent with IDE's strong cleavage-site preferences at P_1_' and P_2_' and weak preferences at P_3_' and P_4_' (Fig. 1A).

Second, the active site of IDE is configured such that the P_1_' and P_2_' residues of Ii1 both point away from IDE's internal chamber (Fig. 4A,B), thereby inducing a partial turn within the amide backbone of Ii1. This partial turn, which is also present within several full-length IDE substrates [9], appears to be a structural prerequisite permitting extended peptides to fit within IDE's internal chamber. This configuration would appear to disfavor hydrolysis of substrates that extend from the inside to the outside of the protease, a feature that likely accounts for IDE's inability to process larger protein substrates and its lack of exopeptidase activity.

Finally, and quite significantly, Ii1's hydroxamate moiety, amide backbone, and P_2_' residue all make substantial interactions not only with residues in the N-terminal portions of IDE involved in zinc-binding and catalysis, but also with multiple residues within distal C-terminal portions of the protease (Fig. 4B; Fig. S4). This finding highlights the fact that IDE's active site is not completely formed unless and until the N- and C-terminal portions of the protease are juxtaposed, which occurs only in the closed conformation and only during a portion of IDE's catalytic cycle [9], [18]. Consequently, Ii1 appears to inhibit IDE not only by active-site blockade but also by the novel mechanism of locking IDE in the closed, inactive conformation. Together, these atypical attributes of IDE's active site and its catalytic mechanism may account for the long-standing elusiveness of small-molecule IDE inhibitors, while at the same time auguring well for the eventual development of highly selective compounds (see *Discussion*).

### Unexpected potency of derivatized IDE inhibitors

Because retro-inverso peptide hydroxamates could be generated with facility by solid-phase peptide synthesis, we explored the development of affinity labeled versions containing benzoylphenylalanine at either the P_2_' or P_3_' position together with a fluorescent EDANS label, and in some cases, a biotin group attached via a polyethylene glycol linker. The biotinylated versions avidly labeled recombinant IDE, but showed rather broad cross-reactivity in cell lysates under the conditions tested (not shown), probably due in part to their very large size (∼1600 Da). Surprisingly, however, the addition of these extraneous functionalities resulted in considerable increases in potency, with *K*_i_ values in the range of 0.3 to 4 nM (Table S4), an effect that we speculate may relate to a reduced off rate due to entrapment within IDE's internal chamber. Importantly, this effort resulted in the generation of 4-amino acid retro-inverso compounds that, relative to Ii1, were not only comparable in size and potency but also contained divergent residues at all positions but P_1_' (*e.g.*, ML3-XF; Table S4). Moreover, the inverted peptide backbone in these compounds is predicted to render them more resistant to degradation than conventional peptidic inhibitors, and they proved useful in downstream cellular assays.

### Effects on catabolism of extracellular and prebound insulin

**Figure 5:**
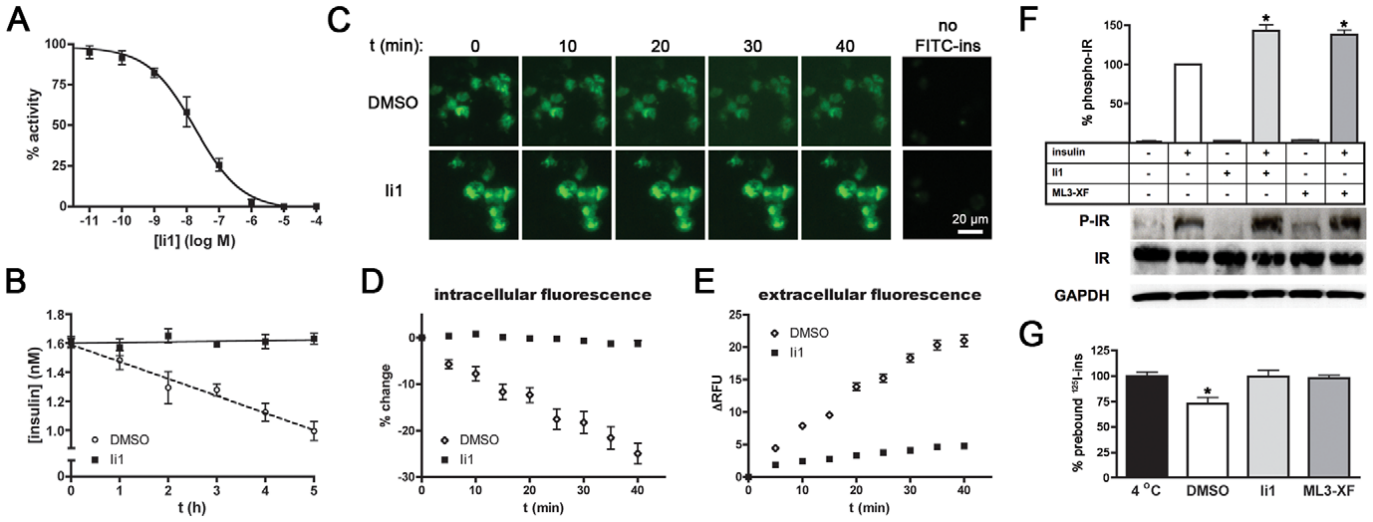
Effects of IDE inhibitors on insulin catabolism and signaling in cells. ***A***, Dose-response curve of Ii1-mediated inhibition of insulin degradation by CHO-IR cells. Note that the potency of Ii1 in this context is comparable to that obtained *in vitro* (*c.f.*, Fig. 3A). ***B***, Progress curve of insulin degradation by CHO-IR cells in the absence or presence of Ii1 (10 µM). Note that insulin catabolism is completely inhibited by Ii1. ***C–F***, Effects of Ii1 on insulin catabolism in live cells. ***C***, Representative images of live CHO-IR cells pre-loaded with FITC-ins and imaged at various time points in the presence or absence of Ii1 (10 µM). ***D,E***, Quantitative analysis of intracellular (***D***) and extracellular (***E***) FITC fluorescence from 5 replicate experiments. Data are mean ± SEM. Note that these experiments were conducted at 22°C. ***F***, Effects of Ii1 on insulin signaling in CHO-IR cells cold-loaded with insulin. Graph shows IR autophosphorylation (phospho-IR) determined by ELISA in response to 5-min incubation at 37°C in the presence of Ii1 (10 µM), ML3-XF (10 µM) or vehicle (DMSO). Data are mean ± SEM of 6 independent experiments, where data are normalized to IR autophosphorylation obtained for insulin and vehicle alone. *p<0.01. Western analysis of a representative experiment (*lower panels*) providing biochemical confirmation of the results obtained by ELISA. ***G***, Preloaded insulin is rapidly catabolized by CHO-IR cells and blocked by IDE inhibitors. Graph shows amounts of ^125^I present in CHO-IR cells cold-loaded with ^125^I-insulin then incubated for 5 min at 37°C in the presence of IDE inhibitors (10 µM) or vehicle then washed to remove insulin catabolites. Data are mean ± SEM of 4 experiments expressed as a percentage of ^125^I present in control cells maintained at 4°C.

To assess the utility of these novel IDE inhibitors as chemical probes, we examined their effects on a range of endpoints relevant to insulin catabolism and signaling in cultured cells. In CHO cells overexpressing the human insulin receptor (IR) (CHO-IR cells) (Fig. 5A) and HeLa cells (Fig. S5A), Ii1 inhibited the degradation of extracellular insulin dose-dependently, with IC_50_s comparable to that obtained *in vitro* (*c.f.*, Fig. 3A). Notably, at µM concentrations, insulin catabolism was inhibited essentially completely (Fig. 5B; Fig. S5B), consistent with other evidence implicating IDE as the major extracellular insulin protease [5]. No toxicity was observed for Ii1 or ML3-XF up to 100 µM (Fig. S6).

Because IDE is in part a secreted protease [24], its role in degrading extracellular insulin is not surprising. By contrast, the extent to which IDE participates in the degradation of internalized insulin remains controversial. The prevailing dogma suggests that, following binding to the IR, insulin is rapidly endocytosed and then degraded in acidic compartments of the endolysosomal system [25]. Consistent with this view, the aspartyl protease cathepsin D has been implicated in the degradation of internalized insulin [26]. On the other hand, IDE has been reported to be present in endosomes, where it may degrade insulin at neutral pH [27]. The development of Ii1—which potently inhibits IDE, but not cathepsin D (Fig. 2C)—enabled us for the first time to address cleanly this longstanding controversy. To that end, we conducted live-cell imaging of CHO-IR cells loaded with fluorescent insulin labeled exclusively at the N-terminus of the B chain with fluorescein isothiocyanate (FITC-ins), a modification that has been shown not to interfere with binding to the IR [28]. FITC-ins-loaded cells were washed then monitored for changes in fluorescence in the presence of Ii1 or vehicle (Fig. 5C). In vehicle-treated cells, intracellular fluorescence decreased (Fig. 5D) and extracellular fluorescence increased (Fig. 5E) monotonically with time. By contrast, both intra- and extracellular fluorescence remained essentially constant in the presence of Ii1 (Figs. 5D,E). Consistent with previous studies of insulin catabolism[29], the fluorescent species secreted by vehicle-treated cells were confirmed to be proteolytic fragments of FITC-ins (Fig. S7). These results strongly suggest that the catabolism of internalized insulin is primarily, if not exclusively, carried out by IDE.

### Unanticipated effects of IDE inhibitors on insulin signaling

Given the historic interest in IDE inhibition as a potential antidiabetic treatment, we assessed the effects of our novel inhibitors on insulin signaling. To simplify interpretation, we focused on the most upstream event in the insulin signaling cascade: IR autophosphorylation. To that end, CHO-IR cells were loaded with insulin at 4°C and washed extensively (in the absence of inhibitors), then warmed to 37°C for 5 min in the presence of IDE inhibitors (10 µM) or vehicle alone. The degree of insulin-stimulated (but not basal) IR autophosphorylation was consistently potentiated in the presence of Ii1 or the retro-inverso IDE inhibitor, ML3-XF (Fig. 5F; Table S4). Though surprisingly brief, the time-scale of this effect is consistent with classic studies of insulin degradation showing that the prebound pool of insulin is degraded very rapidly [29]. To verify this in our experimental system, we directly quantified the degree of degradation of ^125^I-insulin prebound to CHO-IR cells. In good agreement with previous studies [29], prebound ^125^I-insulin decreased ∼30% within 5 min at 37°C in vehicle-treated cells, an effect that was inhibited completely by Ii1 and ML3-XF (Fig. 5G).

Taken together, these results suggest that IDE normally regulates insulin signaling by virtue of its ability to rapidly degrade internalized pools of insulin; correspondingly, insulin signaling can be potentiated significantly by inhibiting IDE proteolytic activity. In addition to illustrating the utility of our novel compounds as experimental probes, these findings lend fresh support to the long-standing prediction that IDE inhibitors could hold therapeutic potential as primary or adjunct treatments for diabetes.

## Discussion

Here we describe the rational design, synthesis, enzymologic characterization, and co-crystallographic analysis of potent and selective peptide hydroxamate inhibitors of IDE. In addition, we use these compounds to show that IDE regulates fundamental aspects of insulin catabolism and signaling in a manner that implies that IDE inhibitors could have anti-diabetic properties. Although the inhibitors described in this study are unlikely to have immediate value as therapeutic agents due to their peptidic nature, their development and the chemical biology they make possible are significant in several important respects.

First and foremost, these compounds constitute the first potent and selective inhibitors of IDE or, indeed, of *any* member of the inverzincin superfamily of zinc-metalloproteases [16]. Given the longstanding interest in IDE in general, and the predicted therapeutic value of IDE inhibitors in particular, why has their development proved so elusive for so long? The answer can be traced to the distinctive structure of IDE's active site, which in turn reflects the separate evolutionary origins of this protease superfamily [7]. As documented by earlier studies [9], [18] and the present work, IDE's active site is bipartite, consisting of two distinct domains contained within the C- and N-terminal halves of the protease. The active site becomes fully formed only when the protease is in the closed conformation, and it is disrupted completely upon transition to the open conformation. These very large conformational changes occurring during the catalytic cycle of IDE essentially render its active site a “moving target,” one that cannot easily be stably occupied by small molecules, even those containing a strong zinc-binding moiety. As our co-crystal structure reveals, the potency of Ii1 can be traced to its unique ability to interact simultaneously with both the N- and C-terminal portions of the active site. In so doing, Ii1 appears to “lock” the protease in the closed, inactive conformation—a feature that is likely to be indispensable for effective IDE inhibitors.

Second, these IDE inhibitors grant several new insights into the enzymology of this poorly understood protease. A particularly puzzling property is the substrate-dependence of *K*_i_ values for inhibition of IDE by Ii1, wherein smaller substrates show lower *K*_i_ values than larger substrates. These two categories of substrate have in fact been shown to exhibit strikingly different behaviors in multiple contexts. For example, the hydrolysis of short substrates—but not longer ones—can be profoundly activated by ATP and other nucleotide polyphosphates [30], [31], inorganic triphosphate [30], [31], as well as by structurally unrelated drug-like molecules [23]. In terms of the differences in *K*_i_ values, we speculate that larger substrates may be more capable than smaller ones of effecting the transition between the closed and open configurations, resulting in an increased off rate for the inhibitor. It may also be that the inhibitor can be trapped inside the internal chamber only in the case of smaller substrates [23]. Alternatively, given that 2 residues within Ii1 protrude into the internal chamber, it may be that larger substrates sterically block a subset of binding modes of the inhibitor. In this context, it is relevant to note that larger substrates are known to interact with an exosite present inside the catalytic chamber but opposite to the active site [9]. It is conceivable that larger substrates utilize this exosite as a point of leverage for larger substrates that normally helps position the substrate into the active site, but which may at the same time have the effect of pushing out an inhibitor. The finding that Hill slopes were consistently <1 is also noteworthy. Given that IDE normally exists as a homodimer [32], [33], this may be explained by intersubunit interactions, wherein the closing of one subunit, for example, by binding to inhibitor, favors the opening of the other subunit. Consistent with this idea, Song *et al.* recently reported [34] that a homodimerization-deficient IDE mutant exhibits markedly different enzymological properties than normal homodimeric IDE. For example, this mutant is not activated by polyphosphates or by other substrates [34]. Together with our own findings, these results suggest that intersubunit interactions powerfully influence the activity and substrate selectivity of IDE, possibly by influencing the transitions between the closed and open configurations of each subunit. Yet another interesting enzymological point emerges from the dose-response studies: the range of doses tested overlapped the nominal enzyme concentration (1 nM); nevertheless, the sigmoidal shape of the corresponding dose-response curves implies a much smaller amount of active enzyme. Consistent with the conclusions of previous studies [9], [31], this result suggests that the vast majority of the protease is normally in the closed, inactive configuration. The finding that only a small fraction of IDE molecules are normally active at any one time may also help to account for IDE's ability to be profoundly activated by multiple ligands [23], [30], [31], [35]. Finally, the observation that Ii1 exhibits a purely competitive mode of inhibition is notable, because it suggests zinc-binding may not be the sole determinant of inhibitor potency; instead, this finding reinforces the idea that the inhibitory power of Ii1 may be better explained by its ability to stabilize the closed, inactive conformation, by virtue of joint interactions with both the N- and C-terminal domains of the protease.

A third significant implication of our study relates to the degree to which Ii1 is selective for IDE *vis-à-vis* other zinc-metalloproteases (∼4 orders of magnitude), which is especially impressive given their peptidic nature. While we emphasize that we cannot exclude the possibility that these compounds cross-react with other zinc-metalloproteases we did not test, it is significant to note that IDE was not inhibited at all even by broad-spectrum hydroxamic acid inhibitors of conventional zinc-metalloproteases (Table S2). These twin findings strongly suggest that it may be possible to develop highly selective IDE inhibitors, even inhibitors containing the potent hydroxamic acid moiety. In this context, it is notable that hydroxamic acids were once considered to be attractive candidates for several therapeutic applications and, indeed, continue to be tested in human trials [36]; however, as a general class, hydroxamic acid protease inhibitors fell out of favor due to a series of disappointing clinical results [37], which are commonly attributed to an innate lack of selectivity of the hydroxamic acid moiety. The remarkable degree of selectivity observed for Ii1 supports the alternative interpretation that the aforementioned clinical failures might instead be attributed to liabilities inherent in the *targets* of the tested compounds—more specifically, to the high degree of structural relatedness and sheer number of conventional zinc-metalloproteases present in higher mammals. Given the marked evolutionary and structural divergence of the inverzincin superfamily, and the low number of its membership, we speculate that it may be feasible to develop hydroxamate inhibitors of IDE with far fewer off-target effects. We emphasize, however, that it should also be possible to develop effective IDE inhibitors containing alternative zinc-binding moieties.

Fourth, and perhaps most significantly from a biomedical perspective, the IDE inhibitors we developed show novel and potentially medicinally valuable effects on insulin signaling. Whereas IDE inhibitors have long been predicted to slow the catabolism of circulating insulin [38], we show here that IDE inhibitors also potentiate the action of insulin subsequent to its binding to the IR. Notably, the latter effect occurs on a time scale that is inconsistent with effects on extracellular insulin catabolism, but instead appears to be attributable to decreased degradation of internalized insulin, which is normally rapidly catabolized [29]. Thus, IDE inhibitors with appropriate pharmacokinetic properties may be useful in combating diabetes by virtue of both “insulin sparing” and “insulin sensitizing” effects. It is worth noting here that the wisdom of inhibiting IDE has been called into question by results from mice genetically deleted for the IDE gene [5]. In particular, IDE knockout mice have themselves been shown to develop a diabetic phenotype [5]. However, it is critical to recognize that this phenotype is a compensatory change that emerges only as a secondary consequence of chronic hyperinsulinemia (S. Abdul-Hay, A. DelleDonne, L. Li, J. Zhao & M. Leissring, unpublished observations; see also [5]). In contrast to the lifelong, pancellular deletion of IDE present in these animals, drugs that only partially or transiently block IDE activity and/or target only a subset of IDE molecules would not be expected to give rise to these compensatory changes, but would instead be expected to improve glycemic control [38]. IDE knockout mice also show elevated levels of cerebral Aβ, raising concern that chronic inhibition of IDE could increase the risk for AD [5]. However, pharmacological inhibitors of IDE could easily be engineered to be blood-brain barrier impermeant, thereby obviating this concern. Moreover, in light of the recent finding that intranasal insulin improves cognition in early AD patients [39], and given insulin's beneficial effects on learning and memory [1], it may be overly simplistic to assume that IDE's role in AD pathogenesis is limited to its predicted effects on Aβ alone.

Finally, the inhibitors we have developed constitute important new tools for the experimental manipulation of IDE, tools that are long overdue [16]. In this connection, it is significant to note that EDTA does not inhibit IDE except after prolonged incubation [18], [40]. The use of IDE inhibitors might therefore be critical for many routine experimental as well as clinical applications involving short peptides. Thus, despite their peptidic nature, the IDE inhibitors described herein should be of immediate use for addressing a number of outstanding questions concerning the chemical biology of this important protease, as well as for accurately quantifying its substrates. Moreover, the insights derived from our co-crystal structure, the first IDE-inhibitor complex, provide a key starting point for the development of more drug-like IDE inhibitors. Given that IDE resides predominantly in the cytosol and other intracellular compartments [41], where its function remains ill-defined, the development of cell-permeant IDE inhibitors will be a particularly important goal. Furthermore, the development of IDE inhibitors that are stable and non-toxic *in vivo* will permit a more thorough evaluation of the physiological and pathophysiological roles of IDE proteolytic activity in animal models of disease, which in turn could lead to new treatments for diabetes and other disorders.

## Methods

### Analysis of IDE's cleavage-site specificity

To determine the amino acids preferred by IDE at positions C-terminal to the scissile bond, IDE was allowed to partially hydrolyze a mixture of N-terminally acetylated 12-mer peptides (Ac-XXXXXXXXXXXX-amide, where “X” refers to any amino acid except cysteine). The resulting C-terminal peptide fragments, containing unblocked N-termini, were analyzed by successive rounds of Edman degradation as described [22]. To determine amino acids preferred at positions N-terminal to the scissile bond, the results of the above analysis were used to generate a second peptide mixture (MGXXXXYKPEDK [K-biotin]-amide) designed to promote hydrolysis N-terminal to the sequence YKPE. Following partial hydrolysis with IDE, biotinylated species, comprising intact peptides and C-terminal fragments, were removed with an avidin column, and the remaining peptides, consisting of freshly cleaved N-terminal fragments, were analyzed by Edman degradation.

### Synthesis of conventional peptide hydroxamic acids

The synthesis of the conventional peptide hydroxamic acids is described in Fig. S8. Synthesis of distereomerically pure intermediates used to confirm the stereochemical assignments of the conventional peptide hydroxamic acids is also described in Figs. S9, S10.

### Synthesis of retro-inverso peptide hydroxamic acids

9-fluorenylmethyleneoxy-carbonyl (Fmoc)-based solid-phase peptide peptide synthesis was carried out manually using a 2-chlorotritylhydroxyl amine resin (Sigma-Aldrich). Following deprotection of the resin with 20% piperidine in dichloromethane (DCM), Fmoc-protected β-D-amino acids (5 eq.) were coupled in a 16-hr reaction using HATU (5 eq.), HOAt (5 eq.) and DIEA (10 eq.) in DCM, and subsequent amino acids (4 eq.) were coupled using HBTU (4 eq.), HOBt (4 eq.) and DIEA (4 eq.) in dimethylformamide (DMF). Cleavage from the resin and removal of protecting groups was effected by a mixture of trifluoroacetic acid (95%), triisopropylsilane (2.5%) and water (2.5%).Peptides were purified by reverse-phase liquid chromatography using a linear acetonitrile:water gradient and masses were verified by matrix-assisted laser desorption/ionization time-of-flight (MALDI-TOF) mass spectrometry.

### Crystallization and structural refinement of IDE-Ii1 complex

IDE-CF-E111Q, a catalytically inactive IDE mutant free of cysteines (C110L, C171S, C178A, C257V, C414L, C573N, C590S, C789S, C812A, C819A, C904S, C966N, and C974A) was constructed using the QuickChange Multi Site-Directed Mutagenesis Kit according to manufacturer's recommendations (Stratagene). IDE-CF has catalytic activity comparable to wild-type IDE, and IDE-CF-E111Q readily forms diffracting crystals. Expression and purification of IDE-CF-E111Q were performed as previously described [42]. The complex of CF-IDE-E111Q and the peptide hydroxamate Ii1 was formed by mixing protein and Ii1 in a 1∶1 molar ratio and isolated by a superdex-200 column. Crystals of IDE-Ii1 were grown by hanging drop and cryoprotected as described [42]. Data from a crystal that diffracted to 2.6 Å resolution was collected at 100 K at the Advanced Photon Source Structural Biology Center ID19 (Argonne, IL) and processed with the programs HKL2000/SCALEPACK [43]. Using substrate-free IDE-Y831F (PDB code 2JG4; Ref [31]) as the model in PHASER [44], a satisfactory molecular replacement solution of IDE-Ii1 was obtained. Refinement of the model was performed using anisotropic scaling, bulk solvent correction, rigid body refinement, coordinate and individual restraint *B* factor refinement, and simulated annealing using PHENIX [45]. Building of IDE structure was performed using Coot [46]. Simulated annealing *2F_o_–F_c_* composite omit map was then calculated using CNS [47] and used in modelling Ii1 at the active site. The final model of IDE-Ii1 (PDB code 3E4A) had R_work_ and R_free_ of 16.7% and 22.5%, respectively. The statistics of diffracting data and refinement are summarized in Table S3.

### *In vitro* enzymatic assays

IDE activity was quantified by monitoring hydrolysis of FRET1 [31], SP1 [23], FAβB [17] or ^125^I-insulin [48] as described previously, using recombinant human IDE purified from bacteria [33]. Degradation of unmodified recombinant human insulin (Sigma-Aldrich) and FITC-ins [28] (Invitrogen) was assessed using a homogeneous time-resolved fluorescence (HTRF)-based assay (CIS-Bio). Quantitative kinetic data were derived by hyperbolic regression analysis using the computer program HYPER.EXE created by John S. Easterby (University of Liverpool). Activity of cathepsin B was assessed by monitoring hydrolysis of Z-LR-AMC (Enzo Life Sciences International), that of cathepsin D using the Cathepsin D Assay Kit (Sigma-Aldrich), and that of the 20S proteasome using the 20S Proteasome Assay Kit for Drug Discovery (Enzo Life Sciences International). The activity of all other proteases was assessed by monitoring the hydrolysis of OmniMMP™ Fluorogenic Substrate (Enzo Life Sciences International).

### Cell-based extracellular insulin degradation assays

Recombinant human insulin (Sigma) was applied to CHO-IR cells or HeLa cells grown to near-confluency on 96-well plates under normal cell culture conditions, and its disappearance over time in the presence of different concentrations of IDE inhibitors or vehicle was quantified using a HTRF-based insulin assay (CIS-Bio). CHO-IR cells were a generous gift from Dr. Michel Bernier (National Instutute on Aging) and HeLa cells were purchased from American Type Culture Collection.

### Live-cell imaging of intracellular insulin catabolism

CHO-IR cells were cultured on poly-D-lysine glass bottom culture dishes (MatTek Corp.), loaded with FITC-ins [28] (2 µM; Invitrogen) for 2 h at 37°C, washed with Hank's balanced salt solution (HBSS), then imaged consecutively at fixed intervals by fluorescence microscopy at 22°C. Intracellular and extracellular fluorescence in the resulting images was quantified using MetaMorph software according to manufacturer's recommendations (Molecular Devices).

### IR autophosphorylation assays

CHO-IR cells were grown to near confluency in 6-well plates, washed, then starved for 6 h in insulin-free medium (DMEM/0.1% BSA). Cells were then cold-loaded with human insulin essentially as described [49]. Briefly, after cooling the cells to 4°C, the medium was replaced with ice-cold HBSS/1% BSA supplemented with insulin (10 nM), incubated at 4°C for 1 h, washed 3 times with ice-cold HBSS/1% BSA. After addition of ice-cold HBSS/1% BSA supplemented with IDE inhibitors (10 µM) or DMSO, the cells were incubated at 37°C for 5 min, then rapidly cooled on ice. Cell lysates were harvested using manufacturer-provided cell-lysis buffer (Cell Signaling Technology) supplemented with additional phosphatase inhibitors (Millipore). IR autophosphorylation (Tyr 1146) and total IR levels were quantified by ELISAs (Cell Signaling Technology), and confirmed by western blotting with the same antibodies used for ELISAs or GAPDH as a loading control.

### Quantification of prebound insulin catabolism

Confluent monolayers of CHO-IR cells grown in 6-well culture dishes were cold loaded with 240 pM ^125^I-insulin for 1 h and washed 3 times with ice-cold HBSS/1% BSA. Following addition of ice-cold HBSS/1% BSA supplemented with IDE inhibitors (10 µM) or DMSO, the cells were incubated at 37°C for 5 min (or as a control, maintained at 4°C), then rapidly cooled on ice and washed two times with ice-cold HBSS/1% BSA supplemented with Complete protease inhibitor cocktail (Roche) and Ii1 (10 µM). Following harvesting of cells by scraping, ^125^I levels were quantified by gamma counter (Beckman).

## Supporting Information

Table S1 **Examples of known IDE inhibitors.**
(0.03 MB DOC)

Table S2 **IDE inhibition by commercially available hydroxamic acids.**
(0.05 MB DOC)

Table S3 **Crystallographic statistics.**
(0.07 MB DOC)

Table S4 **Potency of derivatized retro-inverso peptide hydroxamates.**
(0.03 MB DOC)

Figure S1 **IDE inhibitors identified through high-throughput screening of ∼115,000 compounds. A**, Examples of thiol-alkylating compounds, which made up the majority of identified inhibitors. **B**, Compound showing modest inhibition of IDE whose potency was not improved despite extensive medicinal chemistry efforts. **C**, Nullscript, a small-molecule hydroxamic acid, was the only inhibitor apart from thiol-alkylating compounds to show submicromolar potency.
(0.06 MB TIF)

Figure S2 **Structural comparison of conventional (A) and retro-inverso (B) peptide hydroxamates.** Note that, in the retro-inverso compounds, the α-carbon adjacent to the hydroxamic acid moiety requires the use of β-amino acids, and that D-isomers must be used at all positions to mimic the relative orientation of residues in conventional peptides.
(0.06 MB TIF)

Figure S3 **Kinetic of Ii1-mediated inhibition of Aβ degradation.** **A**, Lineweaver-Burk plot of IDE-mediated Aβ degradation in the absence or presence of Ii1 (30 nM). **B**, Quantitative kinetic data derived from **A**. Note pure competitive mode of inhibition. n = 4 replications.
(0.10 MB TIF)

Figure S4 **Surface representation of IDE showing the interior of the catalytic chamber defined by the N- and C-terminal domains.** IDE-N and IDE-C are rotated by −90 degree (*left*) and +90 degree (*right*) with respect to IDE represented in the ribbon diagram above. Surfaces are colored by electrostatic potentials less than 6kT (*red*) or greater than +6kT (*blue*). The catalytic chamber of IDE-Ii1 contained extra electron density in the region previously shown to accommodate the N-terminus of substrates, which interact with the exosite of IDE. This extra electron density was fitted by a tri-alanine peptide. Surface potentials are displayed with Pymol. The monomeric IDE in the ribbon diagram is colored as *green*, *blue*, *yellow* and *red* for domains 1, 2, 3 and 4, respectively. The molecular surface of IDE is color coded by electrostatic potential, as calculated by APBS2. Ii1 and tri-alanine peptide are drawn in *stick* representation. Carbon, nitrogen, and oxygen atoms of Ii1 and the main chains of peptide at the exosite are colored *orange*, *blue*, and *red*, respectively. Figure generated using Pymol.
(1.58 MB TIF)

Figure S5 **Effects of Ii1 on catabolism of extracellular insulin in HeLa cells.- A**, Dose-response curve of Ii1-mediated inhibition of insulin degradation by HeLa cells. Note that the potency of Ii1 in this context is comparable to that obtained *in vitro* (*c*.*f*., Fig. 3A). **B**, Progress curve of insulin degradation by HeLa cells in the absence or presence of Ii1 (10 µM). Note that insulin catabolism is completely inhibited by Ii1.
(0.05 MB TIF)

Figure S6 **Lack of toxicity of IDE inhibitors used in cell-based assays.** **A**, **B**, Potential cytotoxicity of Ii1 and ML3-XF evaluated by lactose-dehydrogenase release (**A**) and (3-(4,5-dimethylthiazol-2-yl)-5-(3-carboxymethoxyphenyl)-2-(4-sulfophenyl)-2H-tetrazolium (MTS) (**B**) conversion assays. Assays were conducted on confluent CHO-IR cells using the CytoTox 96^TM^ and the CellTiter 96® AQueous Non-Radioactive Cell Proliferation Assay (ProMega) according to manufacturer's recommendations. Essentially identical results were obtained with HeLa cells (not shown).
(0.12 MB TIF)

Figure S7 **Confirmation that FITC-labeled species secreted by FITC-insulin-loaded CHO-IR cells are predominantly breakdown products.** **A**, Relative fluorescence intensity of conditioned medium (CM) from FITC-insulin-loaded cells, unconditioned medium, CM from unloaded cells, and-as a key control-the latter medium supplemented with intact FITC-insulin such that the fluorescence is equivalent to that in the CM from FITC-insulin-loaded cells. **B**, Levels of intact insulin present in the latter samples quantified using a homogeneous time-resolved fluorescence-based assay (CIS-Bio). Note that levels of intact insulin are greatly reduced in the CM from FITC-insulin-loaded cells as compared to that in CM from unloaded cells supplemented with a fluorescent equivalent of intact insulin. **C**, Surface-enhanced laser desorption/ionization-time of flight (SELDI-TOF) spectra from CM supplemented with intact FITC-insulin (upper panel) or CM from FITC-insulin-loaded cells (lower panel). Note that intact FITC-insulin (expected mass = 6197.4; observed mass 6196.37) is detectable in the former sample, but only fragments thereof are detected in the latter.
(0.35 MB TIF)

Figure S8 Synthetic scheme for inhibitor Ii1. a) 10% Pd/C, H_2_ (1 atm), aqueous NaOH, EtOH, rt, 48 h; b) BnBr, K_2_CO_3_, acetone, reflux, 16 h; c) LDA, THF, −78°C, 1 h then BrCH_2_CO_2_t-Bu; d) TFA - CH_2_Cl_2_ (1∶1), rt, 1 h; e) t-BuONH_2_·HCl, DMF, HBTU, DIPEA; f) 10% Pd/C, H_2_ (1 atm), EtOH; g) EtO(C = O)Cl, Et_3_N, THF/DMF, −20°C, 30 min then NH_2_-Arg(Pbf)-allyl·HCl, Et_3_N, −20°C for 1 h then rt for 16 h; h) chromatography on silica gel; i) Pd(PPh_3_)_4_, morpholine; j) HBTU, DIPEA; k) 10% Pd/C, H_2_ (1 atm), EtOH/MeOH; l) 5% anisole in TFA, 40°C, 4.5 h; m) reverse HPLC purification. Hydrocinnamic acid, **2a**, was purchased from Sigma-Aldrich. **2-naphthalenepropanoic acid (2b):** A mixture of 3-(2-naphthyl)acrylic acid (1, 1 g, 0.54 mmol) and 10% Pd-C (100 mg) in ethanol (20 mL) was treated with 20% aqueous NaOH (5 mL) and stirred under hydrogen (1 atm) for 48 h. The mixture was filtered and the filtrate was acidified with 1N HCl and the extracted with EtOAc (250 mL). The organic extract was dried over anhydrous magnesium sulfate, filtered and concentrated to afford **2b** (710 mg, 70%), which was used for the next step without further purification: ^1^H NMR (500 MHz, DMSO-d_6_): δ 2.64 (t, 2H, *J* = 7.8), 2.99 (t, 2H, *J* = 7.8), 7.41–7.49 (m, 3H), 7.72 (s, 1H), 7.82–7.87 (m, 3H), 12.16 (br s, 1H). Procedure for the Preparation of Esters **3a** and **3b** A mixture of the acid (**2a**, 1.5 g, 10 mmole) and potassium carbonate (1.4 g, 10 mmole) in acetone ( 30 ml) was treated with benzyl bromide (1.3 ml, 11 mmole) while stirring at room temperature and heated at reflux overnight. The reaction mixture was cooled, filtered and evaporated to an oil. The resultant oil was purified by silica gel column chromatography with 5% EtOAc in hexane to give **3a** as a colorless oil (1.92 g, 80%):^1^H NMR (500 MHz, CDCl_3_): δ 2.69 (t, 2H, *J* = 8.0), 2.97 (t, 2H, *J* = 7.8), 5.11(s, 2H), 7.18–7.21 (m, 3H), 7.26–7.37 (m, 7H). **3b** (oil, 1.26 g, 87%):^1^H NMR (500 MHz, CDCl_3_): δ 2.79 (t, 2H, *J* = 7.5), 3.15 (t, 2H, *J* = 7.8), 5.12 (s, 2H), 7.27–7.34 (m, 6H), 7.41–7.48 (m, 2H), 7.63 (s, 1H), 7.75–7.82 (m, 3H). Procedure for the Alkylation of Esters **3a** and **3b** To a stirred solution of LDA (2.0 M in THF, 2.7 ml, 5.4 mmole) and anhydrous THF (5 ml) at −78°C was dropwise added ester **3a** (1.28 g, 5.3 mmole) in THF (6 ml). After 1 h at −78°C, *tert*-butyl bromoacetate (0.87 ml, 5.9 mmole) was added neat and the reaction mixture was slowly allowed to warm to room temperature overnight. A saturated aqueous ammonium chloride solution (2 ml) was added to the reaction mixture followed by EtOAc (100 ml). The organic layer was washed with saturated sodium chloride (100 ml), dried over anhydrous sodium sulfate, filtered, evaporated to give an oil. The resultant oil was purified by silica gel column chromatography with 7% EtOAc in hexane to give crude **4a** (1.2 g, 63% mixture of mono- and dialkylated ester) as a colorless oil and used for the next step without further purification. **4b** (oil, 830 mg, 40%):^1^H NMR (500 MHz, CDCl_3_): δ 1.38 (s, 9H), 2.39 (dd, 1H, *J* = 5.0, 16.5), 2.65 (dd, 1H, *J* = 8.8, 17.3), 2.94 (dd, 1H, *J* = 7.5, 13.0), 3.17–3.26 (m, 2H), 5.05 (d, 1H, *J* = 12.0), 5.11 (d, 1H, *J* = 12.5), 7.16–7.19 (m, 2H), 7.22–7.29 (m, 4H), 7.42–7.48 (m, 2H), 7.58 (s, 1H), 7.73–7.76 (m, 2H), 7.78–7.81 (m, 1H). Procedure for the Deprotection of Alkylated Esters **4a** and **4b** to acids **5a** and **5b** A mixture of the ester (**4a**, 1.2 g, 3.78 mmole, crude mixture (see above)) and TFA (10 ml) in dichloromethane (20 ml) was stirred at room temperature for 4 h. The reaction mixture was evaporated to give an oil, which was purified by silica gel column chromatography with 30% EtOAc in hexane to give **5a** as a colorless oil, which solidified under house vacuum (530 mg, 53% from crude starting material):^1^H NMR (500 MHz, CDCl_3_): δ 2.47 (dd, 1H, *J* = 4.5, 17.5), 2.75 (dd, 1H, *J* = 9.5, 17.0), 2.79 (dd, 1H, *J* = 8.5, 13.5), 3.08 (dd, 1H, *J* = 6.3, 13.8), 3.15–3.20 (m, 1H), 5.11 (s, 2H), 7.12–7.13 (m, 2H), 7.19–7.35 (m, 8H). **5b** (waxy oil, 770 mg, 95%):^1^H NMR (500 MHz, CDCl_3_): δ 2.50 (dd, 1H, *J* = 4.8, 17.3), 2.78 (dd, 1H, *J* = 9.0, 17.5), 2.96 (dd, 1H, *J* = 7.5, 13.0), 3.21–3.30 (m, 2H), 5.09 (dd, 2H, *J* = 12.3, 15.3), 7.17–7.19 (m, 2H), 7.22–7.28 (m, 4H), 7.42–7.48 (m, 2H), 7.58 (s, 1H), 7.73–7.76 (m, 2H), 7.78–7.81 (m, 1H). Procedure for the Preparation of Protected Hydroxamates **6a** and **6b** A mixture of the acid (**5a**, 600 mg, 2 mmole) and HBTU (770 mg, 2.02 mmole) in anhydrous DMF (20 ml) was stirred at room temperature for 1 h. Then *O-tert*-butylhydroxylamine HCl (265 mg, 2.1 mmole) and DIPEA (0.74 ml, 2.1 mmole) were added and the resulting reaction mixture was stirred overnight. The reaction mixture was diluted with EtOAc (100 ml), washed with saturated sodium chloride (100 ml), dried over anhydrous sodium sulfate, filtered, evaporated to give an oil. The resultant oil was purified by silica gel column chromatography with 20% EtOAc in hexane to give **6a** (oil, 700 mg, 95%): ^1^H NMR (500 MHz, DMSO-d_6_): δ 1.11 (s, 9H), 2.20 (dd, 1H, *J* = 5.5, 15.0), 2.36 (dd, 1H, *J* = 9.0, 15.5), 2.79–2.80 (m, 2H), 3.07–3.13 (m, 1H), 4.99 (s, 2H), 7.11–7.12 (m, 2H), 7.20–7.35 (m, 8H), 10.36 (s, 1H). **6b** (oil, partially purified, mixture of product and acid-starting material). Procedure for the Preparation of Acids **7a** and **7b** A mixture of hydroxamate (**6a**, 700 mg, 1.89 mmol) and 10% Pd-C (200 mg) in ethanol (20 mL) was stirred under hydrogen (1 atm) for 16 h. The mixture was filtered and concentrated to afford **7a** (white solid, 480 mg, 90%), which was used for the next step without further purification: ^1^H NMR (500 MHz, DMSO-d_6_): δ 1.12 (s, 9H), 2.06 (dd, 1H, *J* = 5.8, 15.0), 2.28 (dd, 1H, *J* = 8.8, 15.3), 2.73 (dd, 1H, *J* = 6.8, 13.3), 2.86 (dd, 1H, *J* = 7.3, 13.3), 2.93–2.98 (m, 1H), 7.16–7.22 (m, 2H), 7.27–7.30 (m, 3H), 10.31 (s, 1H), 12.24 (br s, 1H). **7b** (purified by silica gel column chromatography with 5% MeOH in DCM, white foam, 220 mg, 80%): ^1^H NMR (500 MHz, DMSO-d_6_): δ 1.12 (s, 9H), 2.13 (dd, 1H, *J* = 5.0, 15.0), 2.32 (dd, 1H, *J* = 8.5, 14.5), 2.92 (dd, 1H, *J* = 6.3, 12.8), 3.02–3.08 (m, 2H), 7.35–7.40 (m, 1H), 7.45–7.51 (m, 2H), 7.68 (s, 1H), 7.83–7.89 (m, 3H), 10.32 (s, 1H), 12.30 (br s, 1H). Procedure for the Preparation of Amides **8a–10b** Acid **7a** (140 mg, 0.5 mmole) was dissolved in anhydrous THF (5 ml) and anhydrous DMF (1 ml) and cooled to −20°C. A solution of triethylamine (0.075 ml, 0.54 mmole) in THF (1 ml) was added dropwise to acid **7a**, followed by a solution of ethyl chloroformate (0.048 ml, 0.5 mmole) in THF (1 ml). The reaction mixture was stirred at −20°C for 1h and the treated with NH_2_-Arg(Pbf)-Allyl·HCl ( 380 mg, 0.55 mmole) and triethylamine (0.093 ml, 0.67 mmole) and stirred 1h at −20°C before slowly warmed to room temperature overnight. The reaction mixture was diluted with EtOAc (150 ml), washed sequentially with 0.5N HCl (100 ml), saturated sodium bicarbonate (100 ml), saturated sodium chloride (100 ml) and then dried over anhydrous sodium sulfate, filtered, evaporated to give a white foam. The resultant foam was purified by silica gel column chromatography with 5% MeOH in DCM to give **8a** (foam, 270 mg, 74%). The diastereomers **8a** was separated by silica gel column chromatography with 3% MeOH in DCM to give **9a** (foam, (R)-form, 125 mg, 34%) as first eluting peak: ^1^H NMR (500 MHz, CDCl_3_): δ 1.18 (s, 9H), 1.40–1.80 (m, 4H), 1.46 (s, 6H), 1.66–1.72 (m, 1H), 1.85–1.91 (m, 1H), 2.09 (s, 3H), 2.21–2.24 (m, 1H), 2.52 (s, 3H), 2.53–2.60 (m, 1H, overlap with s), 2.58 (s, 3H), 2.67 (m, 1H), 2.95 (s, 2H), 2.95–2.97 (m, 1H), 3.16 (broad, 2H), 4.45–4.61 (m, 3H), 5.25–5.33 (m, 2H), 5.82–5.92 (m, 1H), 6.35 (broad d, 1H, *J* = 7.5), 6.44 (broad s, 1H), 7.15–7.16 (m, 2H), 7.20–7.23 (m, 1H), 7.26–7.30 (m, 2H), 8.12 (broad s, 1H), while **10a** as second eluting peak (foam, (S)-form, 145 mg, 40%) peak: ^1^H NMR (500 MHz, CDCl_3_): δ 1.21 (s, 9H), 1.30–1.38 (m, 2H), 1.45 (s, 6H), 1.54–1.62 (m, 2H), 1.70–1.78 (m, 1H), 2.09 (s, 3H), 2.35–2.38 (m, 1H), 2.44–2.50 (m, 1H), 2.52–2.58 (m, 1H), 2.53 (s, 3H), 2.58 (s, 3H), 2.78–2.83 (m, 1H), 2.95 (s, 2H), 2.96–2.98 (m, 1H, overlap with s), 3.08 (broad, 1H), 3.15–3.21 (m, 1H), 4.31 (broad, 1H), 4.56–4.62 (m, 2H), 5.23–5.31 (m, 2H), 5.83–5.91 (m, 1H), 6.10 (broad, 1H), 6.34 (broad, 1H), 6.75 (broad, 1H), 7.17–7.20 (m, 3H), 7.24–7.25 (m, 2H), 8.40 (broad s, 1H), The diastereomers **8b** was also separated by silica gel column chromatography with 2.5% MeOH in DCM to give **9b** (foam, (R)-form, 120 mg, 40%) as first eluting peak, peak: ^1^H NMR (500 MHz, CDCl_3_): δ 1.16 (s, 9H), 1.36–1.66 (m, 4H), 1.45 (s, 6H), 1.85 (broad, 1H), 2.08 (s, 3H), 2.23–2.26 (m, 1H), 2.51 (s, 3H), 2.58 (s, 3H), 2.60–2.66 (m, 1H), 2.84–2.88 (m, 1H), 2.93 (s, 2H), 3.10–3.62 (broad m, 4H), 4.34–4.42 (m, 2H), 4.51–4.55 (m, 1H), 5.17–5.24 (m, 2H), 5.69–5.77 (m, 1H), 5.98 (broad, 1H), 6.43 (broad s, 1H), 6.49 (broad d, 1H, *J* = 8.0), 7.29–7.31 (m, 1H), 7.42–7.47 (m, 2H), 7.58 (s, 1H), 7.73–7.80 (m, 2H), 8.36 (broad s, 1H), while **10b** as second eluting peak (foam, (S)-form, 160 mg, 52%) peak: ^1^H NMR (500 MHz, CDCl_3_): δ 1.07 (broad, 2H), 1.20 (s, 9H), 1.43–1.44 (m, 6H), 1.47–1.70 (broad m, 3H), 2.09 (s, 2H), 2.42–2.47 (m, 1H), 2.52–2.58 (m, 1H), 2.52 (s, 3H), 2.58 (s, 3H), 2.88–2.98 (m, 3H, overlap with s), 2.93 (s, 2H), 3.07–3.12 (m, 1H), 3.19–3.23 (m, 1H), 4.26 (broad, 1H), 4.53 (broad d, 2H, J = 6.0), 5.20–5.28 (m, 2H), 5.79–5.85 (m, 1H), 6.26 (broad, 1H), 6.90 (broad, 1H), 7.32–7.34 (m, 1H), 7.37–7.43 (m, 2H), 7.61 (s, 1H), 7.70–7.76 (m, 3H), 8.60 (broad, 1H). Procedure for the Deprotection of Allyl Esters **9a** **–10b** to acids **11a–** **12b** A solution of the allyl ester **9a** (125 mg, 0.17 mmole) in anhydrous THF (20 ml) was treated with tetrakis(triphenylphosphine)palladium(0) (20 mg, 0.017 mmole) and morpholine ( 0.2 ml, 2.29 mmole) at room temperature for 4 h. The reaction mixture was diluted with EtOAc (100 ml), washed with 0.5N HCl (100 ml), saturated sodium chloride (100 ml), dried over anhydrous sodium sulfate, filtered, evaporated to give a waxy solid. The resultant solid was purified by silica gel column chromatography with 5% MeOH in DCM to give **11a** (waxy solid, 60 mg, 51%) and used without further purification. Other Acids **11b** **–12b** were prepared in the same manner with similar yields and used in the next step without further purification. Procedure for the Preparation of Protected Amide **14** The protected dipeptide **14** was obtained as a white foam (1.16 g, 93%) starting from acid **13** and the HCl salt of NH_2_-Glu(tBu)-CONH_2_ following the general procedure described for **6a** and **6b**: ^1^H NMR (500 MHz, DMSO-d_6_): δ 1.39 (s, 9H), 1.61 (s, 9H), 1.70–1.78 (m, 1H), 1.87–1.93 (m, 1H), 2.12–2.22 (m, 2H), 2.90 (dd, 1H, *J* = 10.5, 15.0), 3.09 (dd, 1H, *J* = 3.5, 15.0), 4.18–4.23 (m, 1H), 4.36–4.40 (m, 1H), 4.92–4.98 (m, 2H), 7.10 (s, 1H), 7.22–7.33 (m, 8H), 7.56 (s, 1H), 7.60 (d, 1H, *J* = 8.0), 7.74 (d, 1H, *J* = 8.0), 8.03 (d, 1H, *J* = 8.0), 8.09 (d, 1H, *J* = 8.0). Procedure for the Deprotection of Amide **14** to **15** The deprotected dipeptide **15** was obtained as a white foam (230 mg, 90%) starting from protected amide **14** following the general procedure described for **7a** and **7b**: ^1^H NMR (500 MHz, CDCl_3_): δ 1.45 (s, 9H), 1.68 (s, 9H), 1.88 (sextet, 1H, *J* = 7.5), 2.06–2.12 (m, 1H), 2.22 (dt, 1H, *J* = 6.8, 17.0), 2.38 (dt, 1H, *J* = 16.5, 7.5), 2.86–2.90 (m, 1H), 3.33 (dd, 1H, *J* = 3.5, 15.0), 3.75 (dd, 1H, *J* = 4.3, 9.3), 4.39–4.43 (m, 1H), 5.41 (br s, 1H), 6.47 (br s, 1H), 7.23–7.26 (m, 1H), 7.30–7.34 (m, 1H), 7.48 (s, 1H), 7.61 (d, 1H, *J* = 8.0), 7.95 (d, 1H, *J* = 8.0), 8.12 (br s, 1H). Procedure for the Preparation of Protected Tetrapeptide **16a**, **16b**, **17a** and **17b** The protected tetrapeptide **16a** was obtained as a white foam (138 mg, 70%) starting from (R)-acid **11a** and amine **15** following the general procedure described for **6a** and **6b**. See ^1^H NMRs below. Procedure for the Preparation of Deprotected Tetrapeptide **18a**, **18b**, **19a** and **19b** A solution of the protected tetrapeptide **16a** (100 mg, 0.086 mmole) in TFA (6 ml) was treated with anisole (0.3 mg, 2.76 mmole) and stirred at 40°C for 4.5 h. The reaction mixture was cooled and the volatiles were removed *in vacuo*. The resultant oil was washed with ether (10 ml), dissolved in water (5 ml) and filtered. The filtrates were lyophilized to give white powders that were further purified by reverse-phase liquid chromatography using a linear acetonitrile:water gradient to give **18a** as a white powder: ^1^H NMR (500 MHz, DMSO-d_6_): δ 1.42–1.53 (m, 3H), 1.65–1.77 (m, 2H), 1.86–1.93 (m, 2H), 2.16–2.20 (m, 2H), 2.26 (dd, 1H, *J* = 9.8, 15.3), 2.45–2.47 (m, 1H, overlap with DMSO), 2.80 (dd, 1H, *J* = 6.0, 13.5), 2.96–3.06 (m, 4H), 3.14 (dd, 1H, *J* = 4.8, 14.3), 4.16–4.24 (m, 2H), 4.50–4.54 (m, 1H), 6.76 (broad, 1H), 6.96 (t, 1H, J = 7.5), 7.04 (t, 1H, J = 7.8), 7.09–7.24 (m, 8H), 7.29 (d, 1H, J = 7.5), 7.32–7.34 (m, 2H), 7.56 (d, 1H, *J* = 8.5), 7.97 (d, 2H, *J* = 7.5), 8.09 (d, 1H, *J* = 7.5), 8.76 (s, 1H), 10.45 (s, 1H), 10.81 (d, 1H, *J* = 2.0), 12.11 (broad s, 1H); MALDI-MS obsd 694.3308, calcd 694.3235 [(M + H)^+^ , M  =  C_33_H_43_N_9_O_8_]. 18b: ^1^H NMR (400 MHz, DMSO-d_6_): δ 1.44–1.56 (m, 3H), 1.68–1.79 (m, 2H), 1.87–1.95 (m, 2H), 2.16–2.23 (m, 2H), 2.28–2.31 (m, 1H, overlap with DMSO), 2.45–2.55 (m, 1H, overlap with DMSO), 2.61–2.64 (m, 1H), 2.94–3.16 (m, 5H), 4.15–4.30 (m, 2H), 4.51–4.59 (m, 1H), 6.80 (broad, 1H), 6.96 (t, 1H, J = 7.2), 7.03 (t, 1H, J = 6.8), 7.09 (s, 1H), 7.14–7.18 (m, 2H), 7.27–7.36 (m, 3H), 7.41–7.47 (m, 2H), 7.56 (d, 1H, *J* = 8.0), 7.65 (s, 1H), 7.74 (d, 1H, *J* = 8.8), 7.80–7.82 (m, 2H), 7.95–8.00 (m, 2H), 8.19 (d, 1H, *J* = 7.6), 8.75 (s, 1H), 10.45 (s, 1H), 10.82 (s, 1H); MALDI-MS obsd 744.3103, calcd 744.3391 [(M + H)^+^ , M  =  C_39_H_45_N_9_O_8_]. **19a**: ^1^H NMR (500 MHz, DMSO-d_6_): δ 1.06–1.09 (m, 2H), 1.14–1.23 (m,1H), 1.46–1.54 (m, 1H), 1.80–1.88 (m, 1H), 1.98–2.05 (m, 1H), 2.14 (dd, 1H, *J* = 4.0, 14.5), 2.22–2.31 (m, 2H), 2.41 (dd, 1H, *J* = 10.5, 15.5), 2.61 (dd, 1H, *J* = 6.5, 13.0), 2.71 (dd, 1H, *J* = 9.0, 13.5), 2.84 (q, 2H, *J* = 7.5), 2.93–2.99 (m, 1H), 3.16–3.24 (m, 2H), 3.76–3.80 (m, 1H), 4.09–4.13 (m, 1H), 4.37–4.41 (m, 1H), 6.78 (broad, 1H), 6.86 (s, 1H), 6.97 (t, 1H, J = 7.3), 7.05 (t, 1H, J = 7.8), 7.13–7.19 (m, 5H), 7.23–7.26 (m, 2H), 7.30–7.33 (m, 3H), 7.55 (d, 1H, *J* = 8.0), 7.77 (d, 2H, *J* = 7.5), 8.25 (d, 1H, *J* = 7.0), 8.34 (d, 1H, *J* = 7.0), 8.95 (s, 1H), 10.70 (s, 1H), 10.77 (d, 1H, *J* = 2.5), 12.13 (broad s, 1H), MALDI-MS obsd 694.3321, calcd 694.3235 [(M + H)^+^ , M  =  C_33_H_43_N_9_O_8_].**19b**: ^1^H NMR (400 MHz, DMSO-d_6_): δ 1.47–1.49 (m, 2H), 1.82–1.91 (m, 1H), 2.01–2.05 (m, 1H), 2.13–2.18 (m, 2H), 2.21–2.30 (m, 2H), 2.42–2.54 (m, 2H, overlap with DMSO), 2.69–2.80 (m, 3H), 2.89–2.94 (m, 1H), 3.04–3.07 (m, 2H), 3.16–3.20 (m, 2H), 3.79–3.84 (m, 1H), 4.09–4.15 (m, 1H), 4.38–4.42 (m, 1H), 6.70 (broad, 1H), 6.88 (s, 1H), 6.97 (t, 1H, J = 7.6), 7.05 (t, 1H, J = 7.2), 7.14–7.19 (m, 2H), 7.30–7.36 (m, 3H), 7.43–7.49 (m, 2H), 7.55 (d, 1H, *J* = 7.6), 7.65 (s, 1H), 7.77 (d, 1H, *J* = 7.6), 7.81–7.86 (m, 3H), 8.25 (d, 1H, *J* = 7.6), 8.39 (d, 1H, *J* = 6.4), 8.95 (s, 1H), 10.70 (s, 1H), 10.78 (s, 1H); MALDI-MS obsd 744.2910, calcd 744.3391 [(M + H)^+^ , M  =  C_37_H_45_N_9_O_8_].
(164 KB TIF)

Figure S9 **Synthetic scheme for generation of diastereomerically pure conventional peptide hydroxamic acids.** **a**) t-BuONH2Â·HCl, HBTU, DIPEA; **b**) LiOH then HCl. Note that these experiments were conducted to confirm the stereochemistry assignments made for each diastereomer **18a**, **18b**, **19a**, and **19b** (see Fig. S8).
(0.05 MB TIF)

Figure S10 **Alternate synthetic scheme for generation of conventional peptide hydroxamic acids.** a) t-BuONH_2_·HCl, HBTU, DIPEA; b) LiOH then HCl; c) EtO_2_CCl, Et3N, −78°C then NH_2_-Arg(Pbf)-allyl·HCl. General Procedure for the Preparation of Protected Hydroxamates **21** and **23** The protected hydroxamate **21** obtained as a colorless oil (290 mg, 88%) starting from (R)-acid **20** following the general procedure described for **6a** and **6b**. Also **23** was obtained as a colorless oil (485 mg, 94%) starting from (S)-acid **22**. **21**: ^1^H NMR (500 MHz, CDCl_3_): δ 1.23 (s, 9H), 2.21–2.51 (m, 1H), 2.37–2.80 (m, 1H), 2.83–2.87 (m, 1H), 3.00–3.04 (m, 1H), 3.25 (broad, 1H), 3.65 (s, 3H), 7.14–7.15 (m, 2H), 7.20–7.23 (m, 1H), 7.27–7.30 (m, 2H), 7.75–7.90 (broad m, 1H). **23**: ^1^H NMR (500 MHz, CDCl_3_): δ 1.19–1.22 (m, 9H), 2.21–2.84 (m, 1H), 2.40–2.84 (m, 1H), 2.99–3.04 (m, 1H), 3.18–3.24 (m, 1H), 3.34 (broad, 1H), 3.66 (s, 3H), 7.28–7.30 (m, 1H), 7.43–7.48 (m, 2H), 7.60 (broad s, 1H), 7.67 (broad, 1H), 7.77–7.82 (m, 3H). General Procedure for the Hydrolysis of Methyl Esters **21** and **23** to acids **(R)-7a** and **(S)-7b** A solution of the methyl ester **21** (335 mg, 1.14 mmole) in methanol (20 ml) and water (3 ml) was treated with LiOH monohydrate (96 mg, 2.28 mmole) in water (5 ml) at O°C for 6 h. The reaction mixture was diluted with water (10 ml), acidified with 1N HCl (to pH 2) and extracted with DCM (50 ml×3). The organic layer was washed saturated sodium chloride (100 ml), dried over anhydrous sodium sulfate, filtered, and evaporated to give a waxy solid. The resultant solid was purified by silica gel column chromatography with 2% MeOH in DCM to give **(R)-7a** (waxy solid, 170 mg, 53%) and used without further purification. Acid **(S)-7b** was in prepared in a similar manner and a similar yield and used without further purification. Procedure for the Synthesis of **10b** The protected dipeptide **10b** was obtained as a white foam (80 mg, 73%) starting from (S)-acid **7b** and Pbf-protected arginine allyl ester HCl salt following the general procedure described for **6a** and **6b**. The TLC profile and ^1^H NMR were the same for this material as those prepared by the separation of diastereomers. Procedure for the Synthesis of **18a** The protected dipeptide **9a** was obtained as a white foam (130 mg, 29%) starting from (R)-acid **7a** and Pbf-protected arginine allyl ester HCl salt following the general procedure described for **6a** and **6b**. The TLC profile and ^1^H NMR were the same for this material as those prepared by the separation of diastereomers. The deprotected **18a** was obtained from **9a** as described above. In addition, this material demonstrated the same *in vitro* activity as the material prepared by the separation of diastereomers.
(0.07 MB TIF)
