# Supplementary material for: Designed Inhibitors of Insulin-Degrading Enzyme Regulate the Catabolism and Activity of Insulin
Source: PLoS One. 2010 May 7;5(5):e10504. doi: 10.1371/journal.pone.0010504 (PMC2866327; doi:10.1371/journal.pone.0010504)
Supplement: Table S1 — Examples of known IDE inhibitors. (0.03 MB DOC) [file pone.0010504.s001.doc]

**Table S1.** Examples of known IDE inhibitors.

| Compound | IC_50_ (μM) | Mode of action | Comments |
| --- | --- | --- | --- |
| 1,10 phenanthroline | 300 | zinc chelation | non-selective |
| N-ethylmaleimide | 220 | thiol alkylation | irreversible, cytotoxic |
| bacitracin | 400 | steric blockage | non-selective, cyclic peptide |
