# Supplementary material for: Designed Inhibitors of Insulin-Degrading Enzyme Regulate the Catabolism and Activity of Insulin
Source: PLoS One. 2010 May 7;5(5):e10504. doi: 10.1371/journal.pone.0010504 (PMC2866327; doi:10.1371/journal.pone.0010504)
Supplement: Table S2 — IDE inhibition by commercially available hydroxamic acids. (0.05 MB DOC) [file pone.0010504.s002.doc]

**Table S2.** IDE inhibition by commercially available hydroxamic acids.

| **Compound** | **Type** | **IDE IC_50_ (μM)** | **best known target** | **target IC_50_ (μM)** |
| --- | --- | --- | --- | --- |
| GM6001 (Galardin) | peptidic | 6 | MMP-8 | 0.0001 |
| TAPI-0 | peptidic | 9 | MMP-13 | 0.0002 |
| TAPI-1 | peptidic | 3 | TACE | 0.1 |
| TAPI-2 | peptidic | 11 | TACE | 5 |
| Nullscript | nonpeptidic | 0.9 | N/A | N/A |
| MMP Inhibitor II | nonpeptidic | >100 | MMP-9 | 0.0027 |
| MMP Inhibitor III | nonpeptidic | >100 | MMP-13 | 0.0001 |
| MMP Inhibitor IV | nonpeptidic | >100 | Multiple MMPs | 0.5 |
| MMP-2/9 Inhibitor II | nonpeptidic | >100 | MMP-2 | 0.017 |
| MMP-2/9 Inhibitor IV | nonpeptidic | >100 | MMP-2 | 0.014 |
| MMP-3 Inhibitor II | nonpeptidic | >100 | MMP-3 | 0.13 |
| MMP-3 Inhibitor VII | nonpeptidic | >100 | MMP-3 | 0.025 |
| MMP-8 Inhibitor I | nonpeptidic | >100 | MMP-8 | 0.004 |
| MMP-9 Inhibitor I | nonpeptidic | >100 | MMP-9 | 0.005 |
| MMP-9/13 Inhibitor I | nonpeptidic | >100 | MMP-9 | 0.0009 |
| MMP-9/13 Inhibitor II | nonpeptidic | >100 | MMP-13 | 0.0013 |
