# Supplementary material for: Designed Inhibitors of Insulin-Degrading Enzyme Regulate the Catabolism and Activity of Insulin
Source: PLoS One. 2010 May 7;5(5):e10504. doi: 10.1371/journal.pone.0010504 (PMC2866327; doi:10.1371/journal.pone.0010504)
Supplement: Table S3 — Crystallographic statistics. (0.07 MB DOC) [file pone.0010504.s003.doc]

**Table S3.** Crystallographic statistics.

|  | IDE + Ii1 |
| --- | --- |
| **Data Collection** |  |
| Beamline | APS 19-ID |
| Wavelength (Å) | 0.97918 |
| Space group | *P*6_5_ |
| Cell dimension(Å) |  |
| *a* | 261.4 |
| *b* | 261.4 |
| *c* | 92 |
| Resolution (Å) | 50-2.6 |
| *R*_merge_ (%)^a, b^ | 9.4 (43.7) |
| I/ ^b^ | 16.5 (2.6) |
| Redundancy^b^ | 3.5 (3.3) |
| Completeness (%)^b^ | 97.2 (91.1) |
| Unique reflections^b^ | 375,603 (106,959) |
| **Refinement** | |
| *R*_work_ (%)^c^ | 16.7 |
| *R*_free_ (%)^d^ | 22.5 |
| No. of |  |
| protein atoms | 16,293 |
| inhibitor atoms | 39 |
| solvent molecules | 372 |
| ligand atoms | 128 |
| metal atoms | 2 |
| B factor (Å^2^) | |
| protein | 40.0 |
| inhibitor | 58.5 |
| solvent | 38.7 |
| Rmsd |  |
| bond lengths (Å) | 0.015 |
| bond angles (^o^) | 1.4 |
| Ramachandran plot (%) |  |
| favorable region | 91.3 |
| allowed region | 8.4 |
| generously allowed region | 0.3 |
| disallowed region | 0.0 |
| PDB accession code | 3E4A |

^a^*R*_merge_ = Σ (*I -* 〈 *I* 〉)/ Σ 〈 *I* 〉

^b^Values in parentheses indicate the highest resolution shell

^c^*R*_work_ = Σ*_hkl_*||F_obs_| - *k* |F_calc_||/ Σ*_hkl_*|F_obs_|

^d^*R*_free_, is the *R*_work_ value for 5% of the reflections excluded from the refinement

^a^ *R*_merge_ = Σ (*I -* 〈 *I* 〉)/ Σ 〈 *I* 〉

^b^ Values in parentheses indicate the highest resolution shell

^c^ *R*_work_ = Σ*_hkl_*||F_obs_| - *k* |F_calc_||/ Σ*_hkl_*|F_obs_|

^d^ *R*_free_, is the *R*_work_ value for 5% of the reflections excluded from the refinement
