# Supplementary material for: Designed Inhibitors of Insulin-Degrading Enzyme Regulate the Catabolism and Activity of Insulin
Source: PLoS One. 2010 May 7;5(5):e10504. doi: 10.1371/journal.pone.0010504 (PMC2866327; doi:10.1371/journal.pone.0010504)
Supplement: Table S4 — Potency of derivatized retro-inverso peptide hydroxamates. (0.03 MB DOC) [file pone.0010504.s004.doc]

**Table S4.** Potency of derivatized retro-inverso peptide hydroxamates.

| **Compound** | **Sequence^a^** | ***K*_i_ (nM)** |
| --- | --- | --- |
| retro-inverso Ii1 | Hx-***2Nap****-Arg-Tyr-Glu*-Ac | 180 |
| ML1-XF | Hx-***2Nap****-Arg-Bpa-*Glu(EDANS)-Ac | 1.2 |
| ML1-XFB | Hx-***2Nap****-Arg-Bpa-*Glu(EDANS)-Glu(PEG-biotin)-Ac | 4.2 |
| ML3-XF | Hx-***2Nap****-Bpa-Tyr*-Glu(EDANS)-Ac | 1.7 |
| ML3-XFB | Hx-***2Nap****-Bpa-Tyr*-Glu(EDANS)-Glu(PEG-biotin)-Ac | 0.3 |

^a^To facilitate comparison to conventional peptide hydroxamates, sequences are listed in reverse order. ß-amino acids are indicated by bold text and D-isomers by italics; Hx=hydroxamic acid; Ac=acetyl group. 2Nap=2-naphthylalanine; Bpa=benzoylphenylalanine; EDANS=5-((2-aminoethyl)amino)naphthalene-1-sulfonic acid; PEG=polyethylene glycol.
