# Supplementary figures and images for: Designed Inhibitors of Insulin-Degrading Enzyme Regulate the Catabolism and Activity of Insulin
Source: PLoS One. 2010 May 7;5(5):e10504. doi: 10.1371/journal.pone.0010504 (PMC2866327; doi:10.1371/journal.pone.0010504)

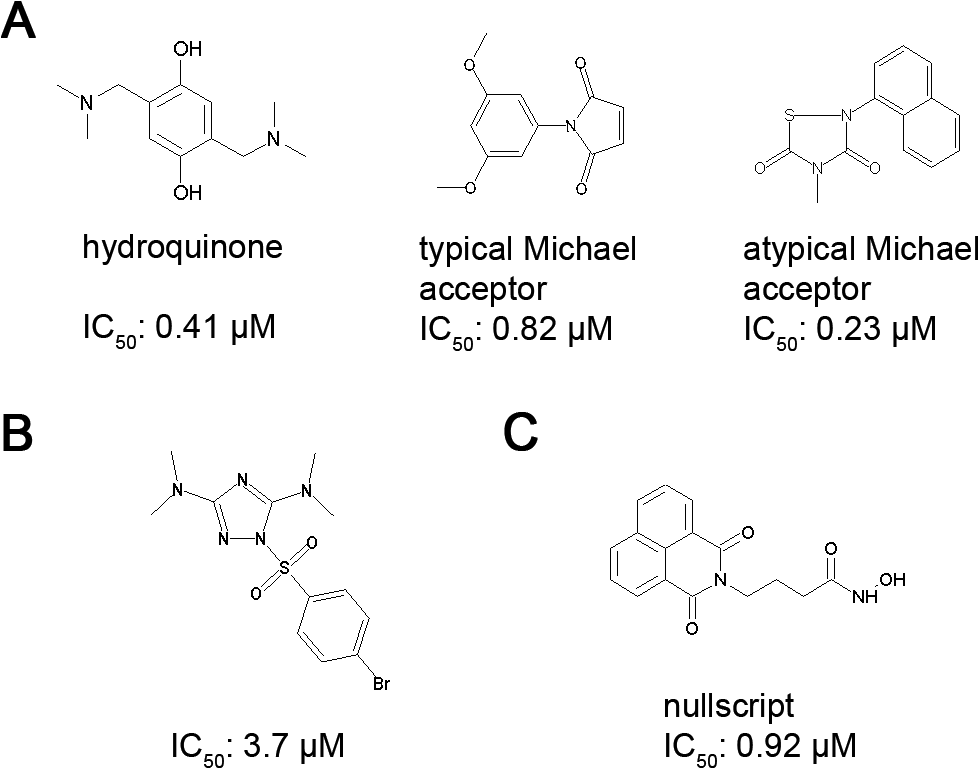

Supplement: Figure S1 — IDE inhibitors identified through high-throughput screening of ∼115,000 compounds. A, Examples of thiol-alkylating compounds, which made up the majority of identified inhibitors. B, Compound showing modest inhibition of IDE whose potency was not improved despite extensive medicinal chemistry efforts. C, Nullscript, a small-molecule hydroxamic acid, was the only inhibitor apart from thiol-alkylating compounds to show submicromolar potency. (0.06 MB TIF) [file pone.0010504.s005.tif]

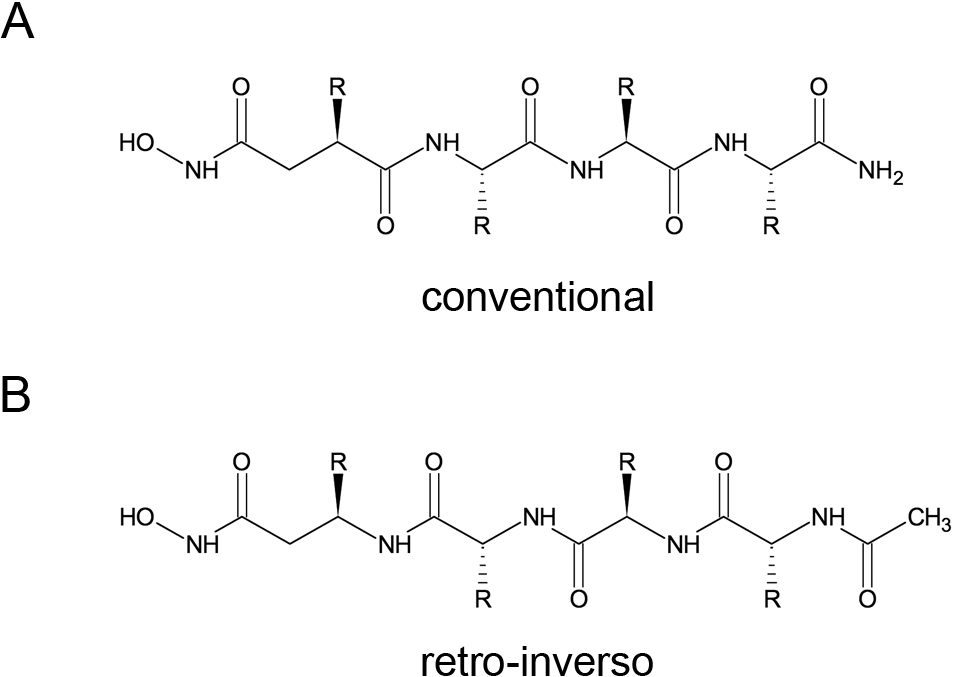

Supplement: Figure S2 — Structural comparison of conventional (A) and retro-inverso (B) peptide hydroxamates. Note that, in the retro-inverso compounds, the α-carbon adjacent to the hydroxamic acid moiety requires the use of β-amino acids, and that D-isomers must be used at all positions to mimic the relative orientation of residues in conventional peptides. (0.06 MB TIF) [file pone.0010504.s006.tif]

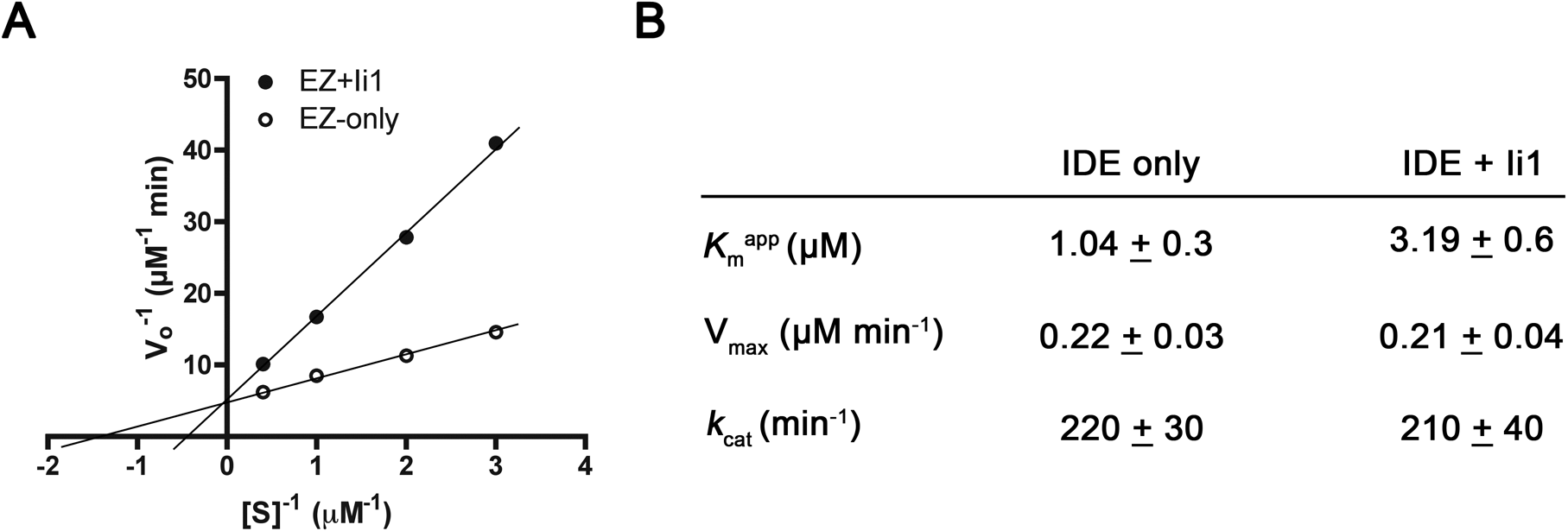

Supplement: Figure S3 — Kinetic of Ii1-mediated inhibition of Aβ degradation. A, Lineweaver-Burk plot of IDE-mediated Aβ degradation in the absence or presence of Ii1 (30 nM). B, Quantitative kinetic data derived from A. Note pure competitive mode of inhibition. n = 4 replications. (0.10 MB TIF) [file pone.0010504.s007.tif]

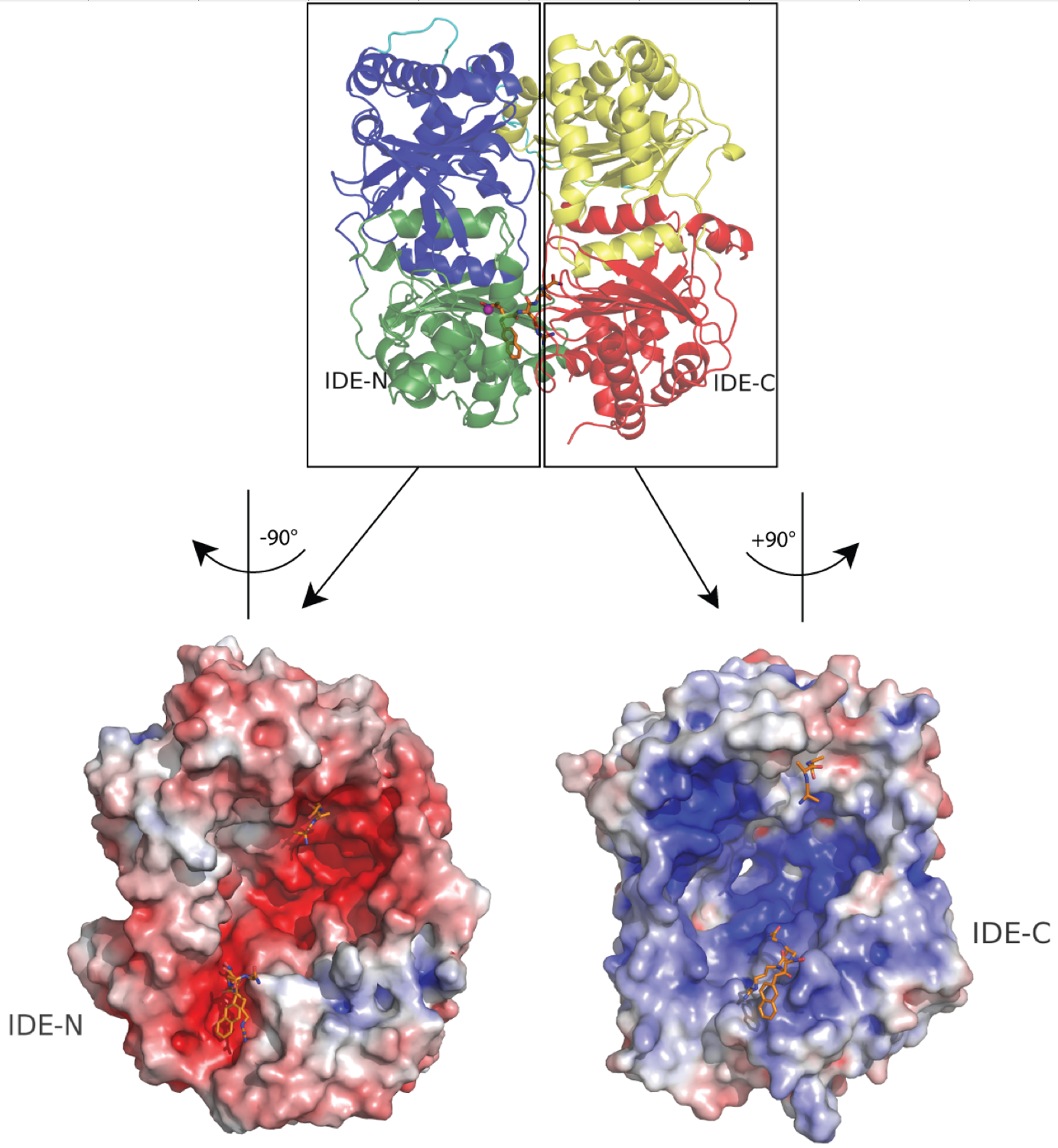

Supplement: Figure S4 — Surface representation of IDE showing the interior of the catalytic chamber defined by the N- and C-terminal domains. IDE-N and IDE-C are rotated by −90 degree (left) and +90 degree (right) with respect to IDE represented in the ribbon diagram above. Surfaces are colored by electrostatic potentials less than 6kT (red) or greater than +6kT (blue). The catalytic chamber of IDE-Ii1 contained extra electron density in the region previously shown to accommodate the N-terminus of substrates, which interact with the exosite of IDE. This extra electron density was fitted by a tri-alanine peptide. Surface potentials are displayed with Pymol. The monomeric IDE in the ribbon diagram is colored as green, blue, yellow and red for domains 1, 2, 3 and 4, respectively. The molecular surface of IDE is color coded by electrostatic potential, as calculated by APBS2. Ii1 and tri-alanine peptide are drawn in stick representation. Carbon, nitrogen, and oxygen atoms of Ii1 and the main chains of peptide at the exosite are colored orange, blue, and red, respectively. Figure generated using Pymol. (1.58 MB TIF) [file pone.0010504.s008.tif]

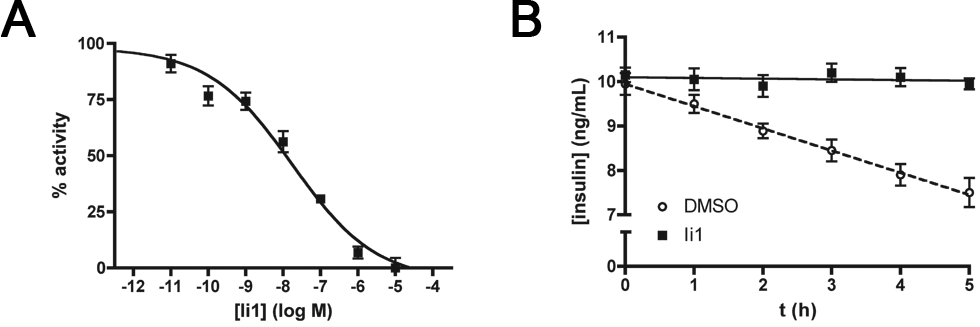

Supplement: Figure S5 — Effects of Ii1 on catabolism of extracellular insulin in HeLa cells.- A, Dose-response curve of Ii1-mediated inhibition of insulin degradation by HeLa cells. Note that the potency of Ii1 in this context is comparable to that obtained in vitro (c.f., Fig. 3A). B, Progress curve of insulin degradation by HeLa cells in the absence or presence of Ii1 (10 µM). Note that insulin catabolism is completely inhibited by Ii1. (0.05 MB TIF) [file pone.0010504.s009.tif]

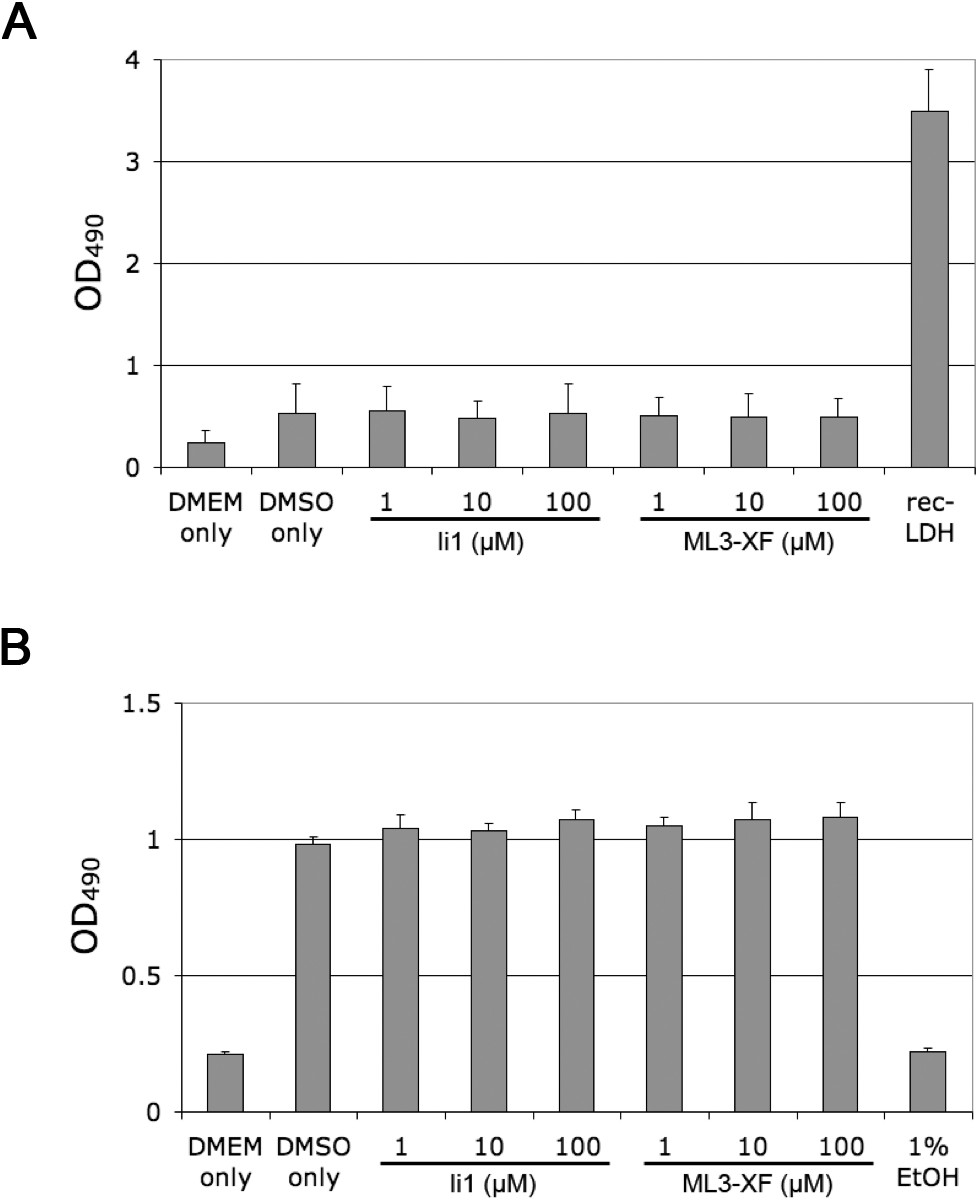

Supplement: Figure S6 — Lack of toxicity of IDE inhibitors used in cell-based assays. A, B, Potential cytotoxicity of Ii1 and ML3-XF evaluated by lactose-dehydrogenase release (A) and (3-(4,5-dimethylthiazol-2-yl)-5-(3-carboxymethoxyphenyl)-2-(4-sulfophenyl)-2H-tetrazolium (MTS) (B) conversion assays. Assays were conducted on confluent CHO-IR cells using the CytoTox 96TM and the CellTiter 96® AQueous Non-Radioactive Cell Proliferation Assay (ProMega) according to manufacturer's recommendations. Essentially identical results were obtained with HeLa cells (not shown). (0.12 MB TIF) [file pone.0010504.s010.tif]

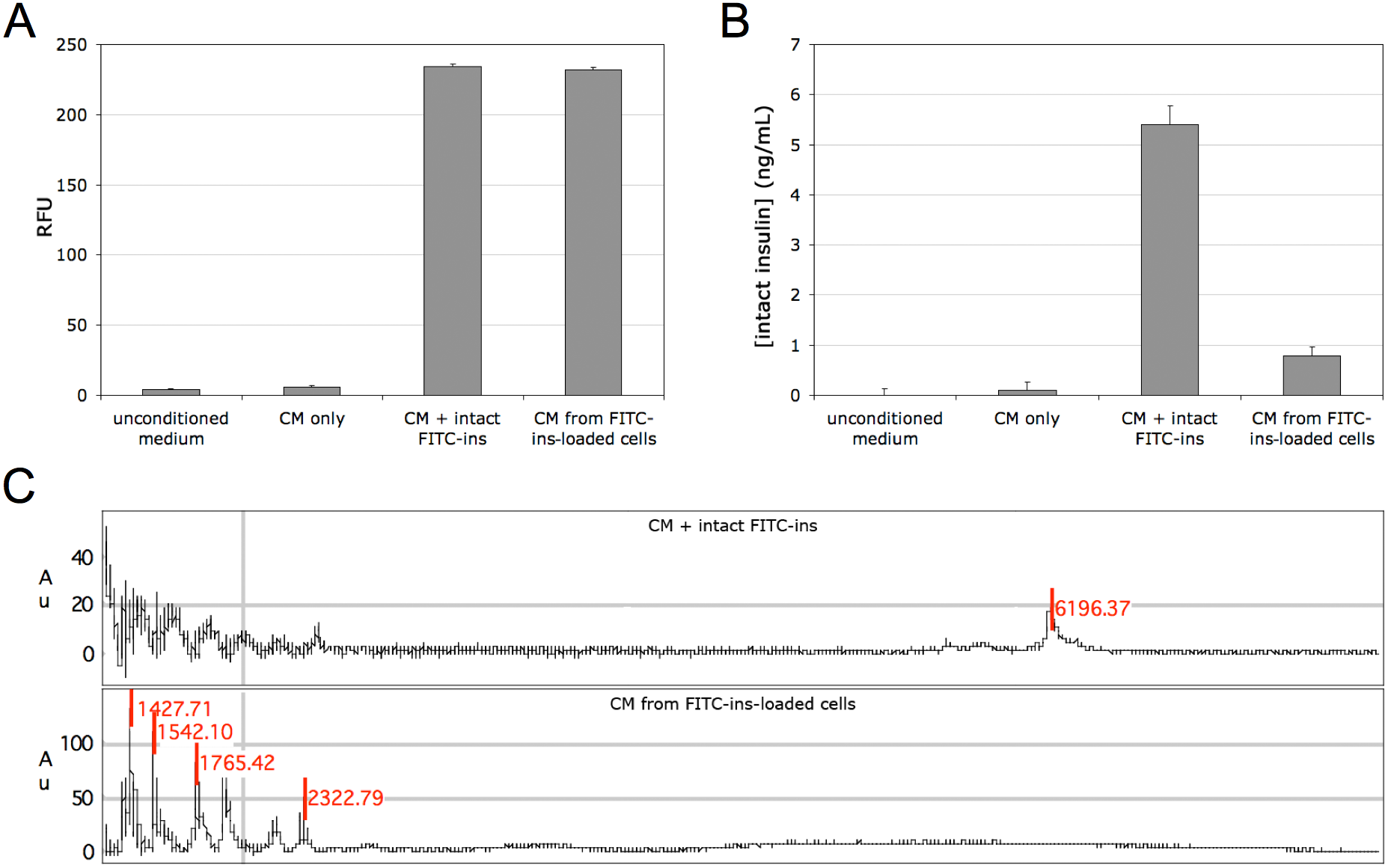

Supplement: Figure S7 — Confirmation that FITC-labeled species secreted by FITC-insulin-loaded CHO-IR cells are predominantly breakdown products. A, Relative fluorescence intensity of conditioned medium (CM) from FITC-insulin-loaded cells, unconditioned medium, CM from unloaded cells, and-as a key control-the latter medium supplemented with intact FITC-insulin such that the fluorescence is equivalent to that in the CM from FITC-insulin-loaded cells. B, Levels of intact insulin present in the latter samples quantified using a homogeneous time-resolved fluorescence-based assay (CIS-Bio). Note that levels of intact insulin are greatly reduced in the CM from FITC-insulin-loaded cells as compared to that in CM from unloaded cells supplemented with a fluorescent equivalent of intact insulin. C, Surface-enhanced laser desorption/ionization-time of flight (SELDI-TOF) spectra from CM supplemented with intact FITC-insulin (upper panel) or CM from FITC-insulin-loaded cells (lower panel). Note that intact FITC-insulin (expected mass = 6197.4; observed mass 6196.37) is detectable in the former sample, but only fragments thereof are detected in the latter. (0.35 MB TIF) [file pone.0010504.s011.tif]

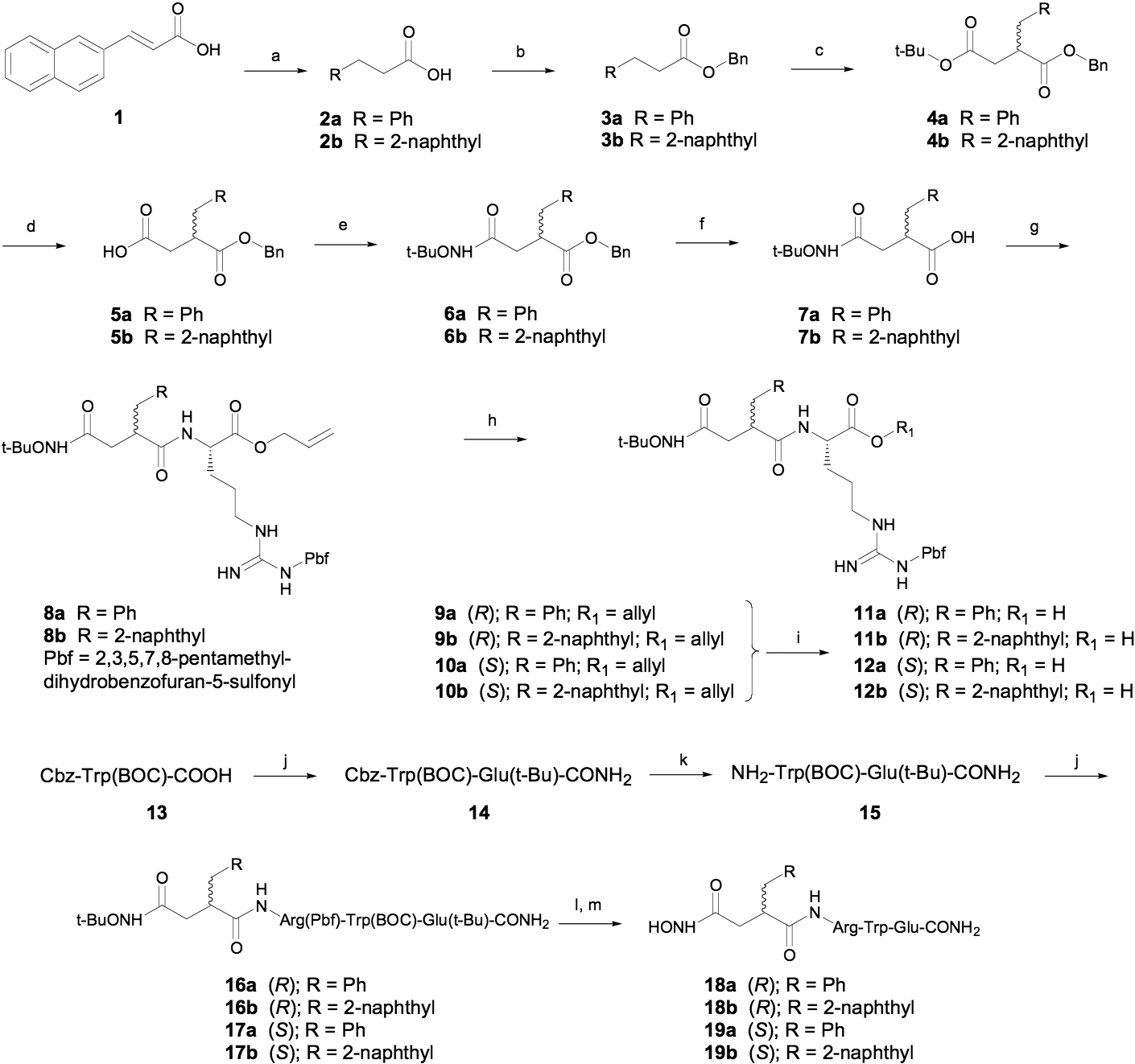

Supplement: Figure S8 — Synthetic scheme for inhibitor Ii1. a) 10% Pd/C, H2 (1 atm), aqueous NaOH, EtOH, rt, 48 h; b) BnBr, K2CO3, acetone, reflux, 16 h; c) LDA, THF, −78°C, 1 h then BrCH2CO2t-Bu; d) TFA - CH2Cl2 (1∶1), rt, 1 h; e) t-BuONH2·HCl, DMF, HBTU, DIPEA; f) 10% Pd/C, H2 (1 atm), EtOH; g) EtO(C = O)Cl, Et3N, THF/DMF, −20°C, 30 min then NH2-Arg(Pbf)-allyl·HCl, Et3N, −20°C for 1 h then rt for 16 h; h) chromatography on silica gel; i) Pd(PPh3)4, morpholine; j) HBTU, DIPEA; k) 10% Pd/C, H2 (1 atm), EtOH/MeOH; l) 5% anisole in TFA, 40°C, 4.5 h; m) reverse HPLC purification. Hydrocinnamic acid, 2a, was purchased from Sigma-Aldrich. 2-naphthalenepropanoic acid (2b): A mixture of 3-(2-naphthyl)acrylic acid (1, 1 g, 0.54 mmol) and 10% Pd-C (100 mg) in ethanol (20 mL) was treated with 20% aqueous NaOH (5 mL) and stirred under hydrogen (1 atm) for 48 h. The mixture was filtered and the filtrate was acidified with 1N HCl and the extracted with EtOAc (250 mL). The organic extract was dried over anhydrous magnesium sulfate, filtered and concentrated to afford 2b (710 mg, 70%), which was used for the next step without further purification: 1H NMR (500 MHz, DMSO-d6): δ 2.64 (t, 2H, J = 7.8), 2.99 (t, 2H, J = 7.8), 7.41–7.49 (m, 3H), 7.72 (s, 1H), 7.82–7.87 (m, 3H), 12.16 (br s, 1H). Procedure for the Preparation of Esters 3a and 3b A mixture of the acid (2a, 1.5 g, 10 mmole) and potassium carbonate (1.4 g, 10 mmole) in acetone ( 30 ml) was treated with benzyl bromide (1.3 ml, 11 mmole) while stirring at room temperature and heated at reflux overnight. The reaction mixture was cooled, filtered and evaporated to an oil. The resultant oil was purified by silica gel column chromatography with 5% EtOAc in hexane to give 3a as a colorless oil (1.92 g, 80%):1H NMR (500 MHz, CDCl3): δ 2.69 (t, 2H, J = 8.0), 2.97 (t, 2H, J = 7.8), 5.11(s, 2H), 7.18–7.21 (m, 3H), 7.26–7.37 (m, 7H). 3b (oil, 1.26 g, 87%):1H NMR (500 MHz, CDCl3): δ 2.79 (t, 2H, J = 7.5), 3.15 (t, 2H, J = 7.8), 5.12 (s, 2H), 7.27–7.34 (m, 6H), [file pone.0010504.s012.tif]

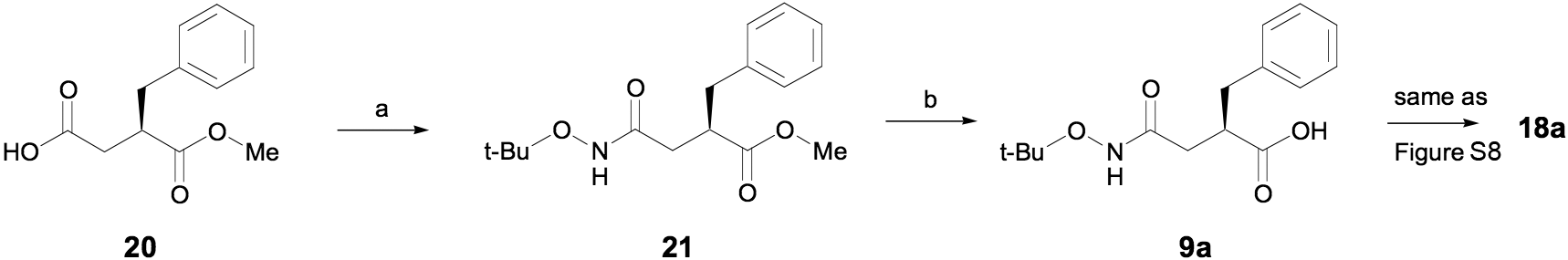

Supplement: Figure S9 — Synthetic scheme for generation of diastereomerically pure conventional peptide hydroxamic acids. a) t-BuONH2Â·HCl, HBTU, DIPEA; b) LiOH then HCl. Note that these experiments were conducted to confirm the stereochemistry assignments made for each diastereomer 18a, 18b, 19a, and 19b (see Fig. S8). (0.05 MB TIF) [file pone.0010504.s013.tif]

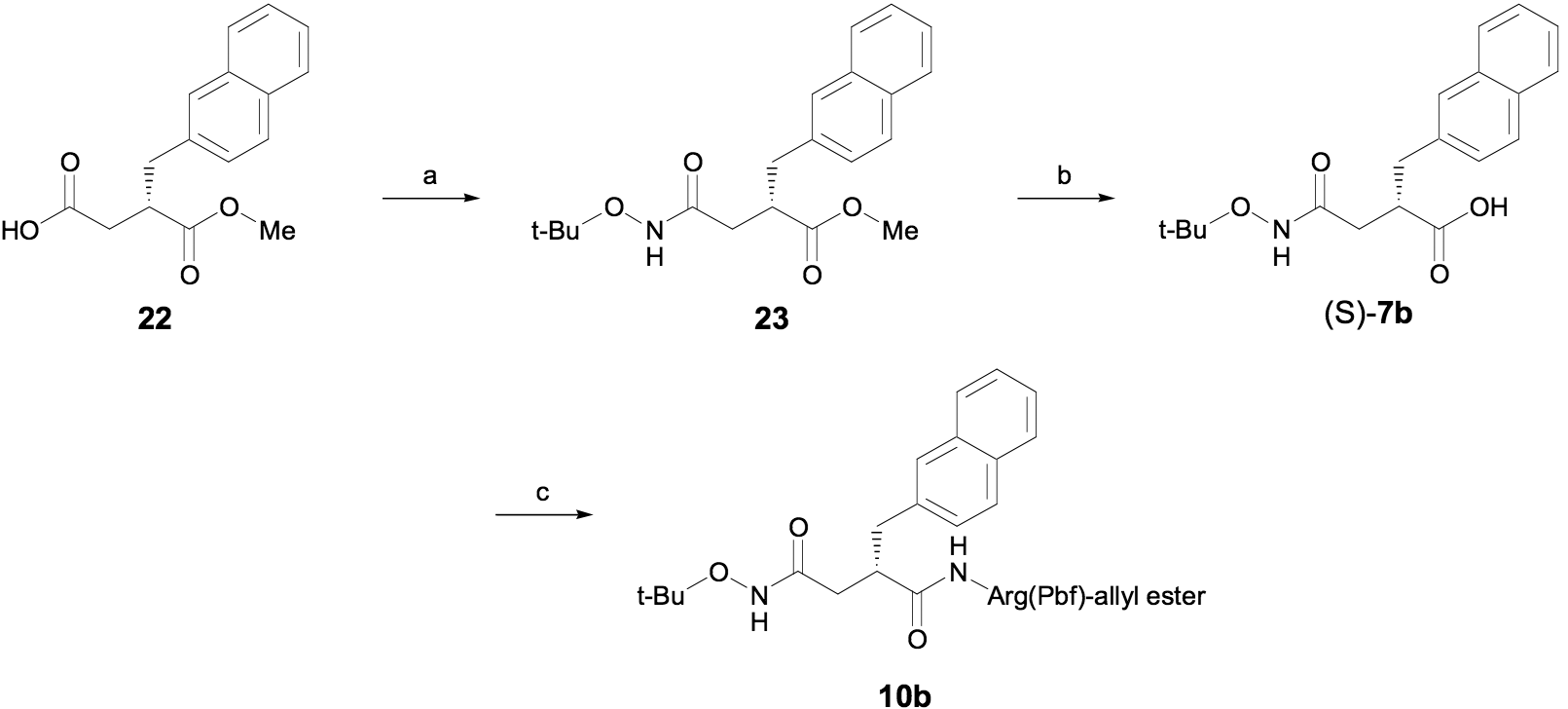

Supplement: Figure S10 — Alternate synthetic scheme for generation of conventional peptide hydroxamic acids. a) t-BuONH2·HCl, HBTU, DIPEA; b) LiOH then HCl; c) EtO2CCl, Et3N, −78°C then NH2-Arg(Pbf)-allyl·HCl. General Procedure for the Preparation of Protected Hydroxamates 21 and 23 The protected hydroxamate 21 obtained as a colorless oil (290 mg, 88%) starting from (R)-acid 20 following the general procedure described for 6a and 6b. Also 23 was obtained as a colorless oil (485 mg, 94%) starting from (S)-acid 22. 21: 1H NMR (500 MHz, CDCl3): δ 1.23 (s, 9H), 2.21–2.51 (m, 1H), 2.37–2.80 (m, 1H), 2.83–2.87 (m, 1H), 3.00–3.04 (m, 1H), 3.25 (broad, 1H), 3.65 (s, 3H), 7.14–7.15 (m, 2H), 7.20–7.23 (m, 1H), 7.27–7.30 (m, 2H), 7.75–7.90 (broad m, 1H). 23: 1H NMR (500 MHz, CDCl3): δ 1.19–1.22 (m, 9H), 2.21–2.84 (m, 1H), 2.40–2.84 (m, 1H), 2.99–3.04 (m, 1H), 3.18–3.24 (m, 1H), 3.34 (broad, 1H), 3.66 (s, 3H), 7.28–7.30 (m, 1H), 7.43–7.48 (m, 2H), 7.60 (broad s, 1H), 7.67 (broad, 1H), 7.77–7.82 (m, 3H). General Procedure for the Hydrolysis of Methyl Esters 21 and 23 to acids (R)-7a and (S)-7b A solution of the methyl ester 21 (335 mg, 1.14 mmole) in methanol (20 ml) and water (3 ml) was treated with LiOH monohydrate (96 mg, 2.28 mmole) in water (5 ml) at O°C for 6 h. The reaction mixture was diluted with water (10 ml), acidified with 1N HCl (to pH 2) and extracted with DCM (50 ml×3). The organic layer was washed saturated sodium chloride (100 ml), dried over anhydrous sodium sulfate, filtered, and evaporated to give a waxy solid. The resultant solid was purified by silica gel column chromatography with 2% MeOH in DCM to give (R)-7a (waxy solid, 170 mg, 53%) and used without further purification. Acid (S)-7b was in prepared in a similar manner and a similar yield and used without further purification. Procedure for the Synthesis of 10b The protected dipeptide 10b was obtained as a white foam (80 mg, 73%) starting from (S)-acid 7b and Pbf-protected arginine allyl ester HCl salt following the general proc [file pone.0010504.s014.tif]
